# Ginger and turmeric expressed sequence tags identify signature genes for rhizome identity and development and the biosynthesis of curcuminoids, gingerols and terpenoids

**DOI:** 10.1186/1471-2229-13-27

**Published:** 2013-02-15

**Authors:** Hyun Jo Koo, Eric T McDowell, Xiaoqiang Ma, Kevin A Greer, Jeremy Kapteyn, Zhengzhi Xie, Anne Descour, HyeRan Kim, Yeisoo Yu, David Kudrna, Rod A Wing, Carol A Soderlund, David R Gang

**Affiliations:** 1 School of Plant Sciences and BIO5 Institute, The University of Arizona, Tucson, AZ, 85721, USA; 2 Arizona Genomics Computational Laboratory and BIO5 Institute, The University of Arizona, Tucson, AZ, 85721, USA; 3 Department of Pharmaceutical Sciences, The University of Arizona, Tucson, AZ, 85721, USA; 4 Arizona Genomics Institute, The University of Arizona, Tucson, AZ, 85721, USA; 5 Institute of Biological Chemistry, Washington State University, Pullman, WA, 99164, USA; 6 Present address: Salk Institute for Biological Studies, PO Box 85800, San Diego, CA, 92186, USA; 7 Present address: XenoBiotic Laboratories, Inc., Morgan Ln 107, Plainsboro, NJ, 08536, USA; 8 Present address: Department of Surgery, College of Medicine, The University of Arizona, Tucson, AZ, 85724, USA; 9 Present address: Division of Cardiovascular Medicine, University of Louisville, Louisville, KY, 40202, USA; 10 Present address: Plant Genome Research Center, KRIBB, Daejeon, 305-803, South Korea; 11 Institute of Biological Chemistry, Washington State University, P.O. Box 646340, Pullman, WA, 99164-6340, USA

**Keywords:** Ginger, Turmeric, EST, Microarray, Metabolite, Rhizome development

## Abstract

**Background:**

Ginger (*Zingiber officinale*) and turmeric (*Curcuma longa*) accumulate important pharmacologically active metabolites at high levels in their rhizomes. Despite their importance, relatively little is known regarding gene expression in the rhizomes of ginger and turmeric.

**Results:**

In order to identify rhizome-enriched genes and genes encoding specialized metabolism enzymes and pathway regulators, we evaluated an assembled collection of expressed sequence tags (ESTs) from eight different ginger and turmeric tissues. Comparisons to publicly available sorghum rhizome ESTs revealed a total of 777 gene transcripts expressed in ginger/turmeric and sorghum rhizomes but apparently absent from other tissues. The list of rhizome-specific transcripts was enriched for genes associated with regulation of tissue growth, development, and transcription. In particular, transcripts for ethylene response factors and AUX/IAA proteins appeared to accumulate in patterns mirroring results from previous studies regarding rhizome growth responses to exogenous applications of auxin and ethylene. Thus, these genes may play important roles in defining rhizome growth and development. Additional associations were made for ginger and turmeric rhizome-enriched MADS box transcription factors, their putative rhizome-enriched homologs in sorghum, and rhizomatous QTLs in rice. Additionally, analysis of both primary and specialized metabolism genes indicates that ginger and turmeric rhizomes are primarily devoted to the utilization of leaf supplied sucrose for the production and/or storage of specialized metabolites associated with the phenylpropanoid pathway and putative type III polyketide synthase gene products. This finding reinforces earlier hypotheses predicting roles of this enzyme class in the production of curcuminoids and gingerols.

**Conclusion:**

A significant set of genes were found to be exclusively or preferentially expressed in the rhizome of ginger and turmeric. Specific transcription factors and other regulatory genes were found that were common to the two species and that are excellent candidates for involvement in rhizome growth, differentiation and development. Large classes of enzymes involved in specialized metabolism were also found to have apparent tissue-specific expression, suggesting that gene expression itself may play an important role in regulating metabolite production in these plants.

## Background

Ginger (*Zingiber officinale* Rosc.) and turmeric (*Curcuma longa* L.) are important not only as spices but also as traditional Eastern medicines for arthritis, rheumatism, fever, nausea, asthma and other ailments [1]. Terpenoids (e.g., turmerones) and phenylpropanoid-polyketides (diarylheptanoids, including the curcuminoids, and the gingerol-related compounds) are believed to be responsible for most of these medicinal properties. Curcumin in particular is used in treatment of cancer, arthritis, diabetes, Crohn’s disease, cardiovascular diseases, osteoporosis, Alzheimer’s disease, and psoriasis, among others [2,3]. [6]-Gingerol also has potential in treating chronic inflammation, such as in asthma and rheumatoid arthritis [4]. This interest in the ginger and turmeric rhizome-associated diarylheptanoids and gingerols has prompted both enzyme assay and metabolic profiling-based inquiries into the biosynthesis of these compounds [2,5-7]. Nevertheless, many of the enzymes involved in production of these compounds in ginger and turmeric have not been identified.

Rhizomes hold greater biological significance as well. The rhizome was the original stem of the vascular plant lineage [8] and is still the only type of stem found in primitive plant groups such as ferns and fern allies. In order to understand the evolution of the upright stem from its rhizomatous origins, we must understand how it differs from the rhizome. Furthermore, we do not understand why and how many advanced plants have “reverted” back to rhizomatous growth. Such reversions have huge economic implications, being responsible for the invasiveness and hardiness of many of the world’s most significant weeds, such as purple nutsedge (*Cyperus rotundus* L.), Johnson grass (*Sorghum halepense* (L.) Pers.), and cogon grass (*Imperata cylindrica* (L.) Beauv.). Thus, increasing our understanding of rhizome biology may have significant impacts not only on our understanding of how important medicinal compounds are produced, but also on our ability to control important weedy species.

Despite the importance of ginger and turmeric and of rhizomes in general, very few genes have been identified from ginger or turmeric rhizomes [9-11]. Moreover, very little is known about the genes involved in rhizome identity, growth and development in general [10-14]. Paterson and coworkers published work on several *Sorghum* species that identified many genes that are expressed in the rhizomes of *S. halepense* and *S. propinquim*[12]. Several of these mapped to QTLs for “rhizomatousness” on the *Sorghum* genetic map. However, the exact role that any of these genes may play in rhizome development remains unclear.

Here we describe the analysis of over 50,000 expressed sequence tags (ESTs) from rhizomes, leaves and roots of two ginger lines (white ginger, GW and yellow ginger, GY) and rhizomes and leaves of one turmeric line (orange turmeric, T3C). Using these ESTs, we identified ginger and turmeric transcripts potentially involved in rhizome biology and specialized metabolism, particularly in the production of curcuminoids, gingerols and terpenoids. Moreover, we provide an explanation for previously observed growth responses of rhizomes to the phytohormones auxin and ethylene [15-18].

## Results and discussion

### Production and analysis of a database of ginger and turmeric ESTs

Random clones from eight cDNA libraries representing rhizome, leaf and root of two ginger lines, and rhizome and leaf of one turmeric line (Additional file 1: Table S1), were 5^′^ and 3^′^ end-sequenced to produce ESTs, which were then assembled into contiguous unique transcriptional units (unitrans) in the Program for Assembling and Viewing ESTs (PAVE, see Methods section). The resulting ArREST (Aromatic Rhizome EST) database (available online at http://www.agcol.arizona.edu/cgi-bin/pave/GT/index.cgi) contains a total of 50,139 ESTs (37,717 from ginger and 12,422 from turmeric) that assembled into 21,215 unigenes (unitrans; 13,717 contigs containing more than one EST and 6,882 singletons). The average EST sequence length was 817 bp, with unitrans lengths ranging from 151 to 4021 bp, with the greatest number of unitrans having between 701 and 800 bp, and with 95% exceeding 300 bp (Additional file 2: Figure S1). Average EST number per unitrans was approximately 3.2, and only fifteen unitrans contained 40 or more ESTs (Additional file 1: Tables S2 and S3), whereas a very large number of the unitrans contained less than 10 ESTs. Many unitrans contained ESTs from both species, suggesting significant homology between these two members of the Zingiberaceae.

Of the 21,215 unitrans identified in the ArREST database, 87.6% could be annotated with Gene Ontologies (GOs). Eight GO categories (Additional file 2: Figure S2) had EST abundances greater than 5%, including protein modification (10.5%), transport (9.2%), metabolism (9.0%), transcription (8.6%), cellular process (8.0%), protein biosynthesis (6.9%), electron transport (5.6%), and biological process unknown (5.3%). Although compounds such as the curcuminoids and gingerols accumulate to high concentration in the rhizome of these plants, the GO category secondary metabolism contained relatively few ESTs (0.3%). Early steps in the pathways to these compounds are covered by other metabolism categories. Other interesting findings from the GO categorization are as follows: 1) transport and metabolism genes appeared to be more highly expressed (based on EST counts) in root than in leaf or rhizome of ginger; 2) genes related to protein modification appeared to be expressed at higher levels in the rhizome than in the leaf or root for both turmeric and ginger; 3) protein biosynthesis genes appeared to be expressed at higher levels in GW roots than other tissues or other plant accessions; and 4) genes classified under the biological process unknown GO category appeared to be expressed at higher levels in turmeric than in ginger.

Based on the GO categorization described above, we were able to outline a metabolic network in ginger and turmeric rhizomes that connects the metabolism of sucrose to the phenylpropanoids and terpenoids (see Additional files 1 and 2: Table S4 and Figure S3). We were also able to analyze the apparent relative expression levels (based on EST abundance) of genes governing the commitment of carbon flux into several primary and specialized metabolic pathways in different tissues (Table 1), as we have previously done for glandular trichomes [19]. These results, which were validated by additional expression studies, suggest that metabolism is regulated in ginger and turmeric rhizomes differently than in leaves or roots, and also support the hypothesis that ginger and turmeric rhizomes are highly specialized for the production of high levels of specialized metabolites.

**Table 1:** Entry point enzymes that regulate carbon partitioning into specific metabolic pathways

**Descriptive name**	**EC #**	**Abbr.**	**T3C**		**GW**			**GY**		
			**Rh**	**L**	**Rh**	**L**	**R**	**Rh**	**L**	**R**
***Primary/core metabolism***										
sucrose synthase	2.4.1.13	SUS	25	0	39	7.1	38	23	14	14
pyruvate kinase	2.7.1.40	KPY	8.8	4.4	16	2.8	6.3	12	0	7.1
pyruvate dehydrogenase	1.2.1.51	PNO	1.8	3.0	6.4	5.7	6.3	3.1	10.1	3.5
***Shikimate pathway***										
DAHP synthetase	2.5.1.54	DAHPS	3.5	0	6.4	0	1.6	14	3.4	7.1
***Phenylpropanoid pathway***										
phenylalanine ammonia lyase	4.3.1.5	PAL	11	0	4.8	16	13	3.1	0	11
***Terpenoid pathway***										
*MEP pathway*										
DOXP reductoisomerase	1.1.1.267	DXR	1.8	0	6.4	0	0	0	0	20
*MVA pathway*										
HMG-CoA reductase	1.1.1.34	HMGR	0	0	0	0	0	0	0	3.5
*Common steps*										
isopentenyl diphosphate isomerase	5.3.3.2	IDI	3.5	0	4.8	1.4	6.3	7.7	0	0
farnesyl diphosphate synthase	2.5.1.10	FPPS	12	8.9	6.4	0	11	34	6.7	11
terpene synthases	N/A	CS	30	7.4	16	0	14	36	0	20
***One carbon metabolism***										
methionine synthase (cobalamin-independent)	2.1.1.14	METE	7	22	18	4.3	17	17	3.4	21

Values given are normalized total EST number (TEN) (×10^4^). TEN, an indicator of EST expression levels, was calculated as the sum of all ESTs that are associated with gene products carrying out a specific enzymatic activity, divided by the total EST number within a particular library.

We also investigated the expression levels for members of eight specific gene families (see Additional file 1: Tables S5 and S6) that play important roles in the biosynthesis of large numbers of specialized metabolites in plants: polyketide synthases (PKSs), terpene synthases (TPSs), NAD(P)H-dependent dehydrogenases/reductases, BAHD acyltransferases, 2-oxoglutarate-dependent dioxgenases (ODDs), SABATH carboxyl methyltransferases, small molecule *O*-methyltransferases (SMOMTs) and cytochrome P450 monooxygenases (P450s). Five of these gene families were particularly well represented in the ArREST database, with normalized total EST numbers of more than 100 for specific family sub-categories. In particular, the P450 gene family was very well represented in the database (see Additional files 1 and 2: Table S6 and Figure S4), suggesting that reactions carried out by members of this family are very important for metabolism in these plants.

### Biosynthesis of diarylheptanoids and gingerols in ginger and turmeric

Of the more than 2,000 nonvolatile compounds detected so far by LC-MS in fresh ginger or turmeric rhizome, less than 100 have been isolated or structurally identified, let alone biosynthetically evaluated [4,20-28]. What is known is that both the diarylheptanoid and gingerol-related classes of compounds are polyketides with origins in the phenylpropanoid pathway [29]. ESTs for phenylpropanoid pathway enzymes were abundant in almost all of the tissues examined. Cinnamate 4-hydroxylase ESTs were the most abundant of the six phenylpropanoid pathway enzymes. Phenylalanine ammonia lyase ESTs, the entry point into the pathway, were abundant in most of the ginger/turmeric cDNA libraries except leaves. Caffeoyl-CoA *O*-methyltransferase (CCOMT) was apparently expressed at higher levels (about 2-fold higher EST counts) in ginger rhizomes than in ginger leaves (Additional file 1: Tables S4 and S5) and was not detectable in turmeric leaf. These results paralleled previous work that showed that CCOMT specific activity was significantly higher in extracts from shoots when compared to leaves and rhizomes for both ginger and turmeric [29], consistent with a role for CCOMT in xylem development. Moreover, the abundance of phenylpropanoid pathway-associated ESTs in rhizomes, which do not accumulate high levels of lignin, flavonoids, lignans, or other phenylpropanoid pathway-derived compounds, supports the hypothesis that the primary biochemical function of ginger and turmeric rhizomes is the conversion of sucrose into the curcuminoids and gingerols [6,7].

**Figure 1:**
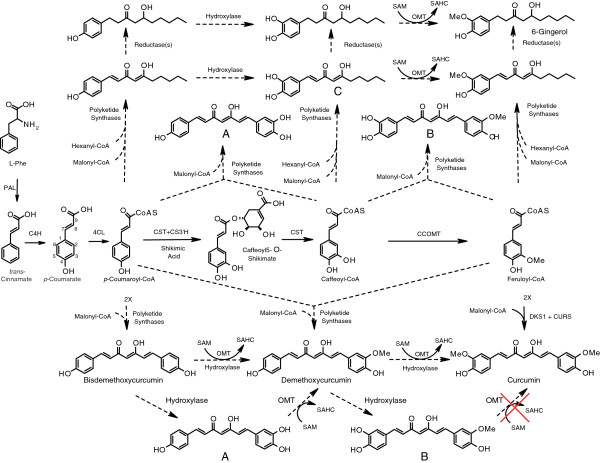
**Proposed biosynthetic pathway from L-Phe to diarylheptanoids and gingerol-related compounds in ginger and turmeric.** Enzymes are as follows: PAL = phenylalanine ammonia lyase; C4H = cinnamate 4-hydroxylase; 4CL = 4-coumarate:CoA ligase; CST = *p*-coumaroyl shikimate transferase; C3´H = *p*-coumaroyl 5-*O*-shikimate 3´-hydroxylase; OMT = *O*-methyltransferase; CCOMT = caffeoyl-CoA *O*-methyltransferase; SAMS = *S-*adenosylmethionine synthetase; SAHC = *S*-adenosylhomocysteine. All conversions have been demonstrated in other species, except for those catalyzed by the polyketide synthases, the reductases, and the hydroxylases and OMTs that would convert bisdemethoxycurcumin via demethoxycurcumin to curcumin (indicated by dashed arrows). The polyketide synthases indicated represent two distinct classes that work in tandem: diketide synthases and curcuminoid/gingerol synthases. Different combinations of these two classes appear to be responsible for production of different compounds in these plants. The large red X and the solid arrows associated with the DKS1 + CURS reactions indicate that formation of curcumin has been demonstrated to proceed directly from feruloyl-CoA, and not through the orthodiol intermediate **B**. Compounds **A** and **B**, therefore, are not likely to be intermediates in the pathway to curcumin but instead are likely to be products of a different pair of PKS enzymes.

**Figure 2:**
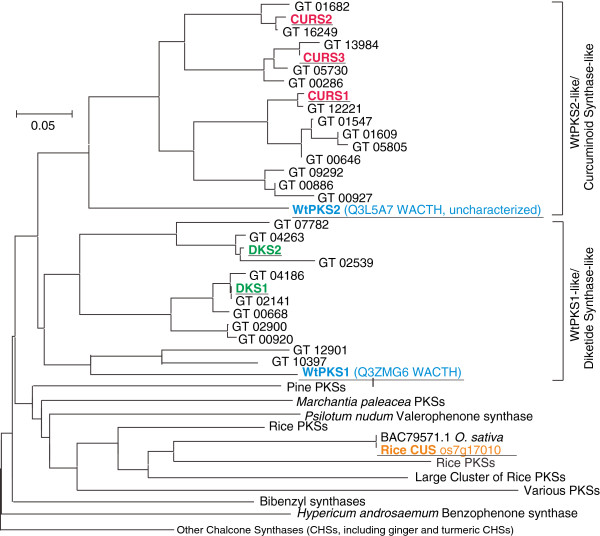
**Ginger and turmeric PKSs that are not bona fide chalcone synthases cluster into two major groups, WtPKS1-like (DKS-like) and WtPKS2-like (CURS-like), in a neighbor-joining similarity tree (parsimony and maximum likelihood approaches produce similar trees).** Abbreviations are as follows: WtPKS, *Wachendorfia thyrsiflora* (Haemodoraceae) polyketide synthase; CURS, turmeric curcumin synthase; DKS, diketide synthass; other PKSs as indicated. In many cases, branches of the tree represent a large number of related sequences, for example, the branch called “*Marchantia paleacea* PKSs” consisted of four closely related sequences, whereas the branch called “Large Cluster of Rice PKSs” represented over 20 sequences, and the branch called “Other Chalcone Synthases” represented over 100 sequences.

Recent metabolic profiling work has suggested that at least two (and probably more) subclasses of polyketide synthases are involved in production of diarylheptanoids in turmeric [6]. One such enzyme can apparently utilize *p*-coumaroyl-CoA and feruloyl-CoA but not caffeoyl-CoA as substrate and produces the major curcuminoids. Additional PKSs can utilize caffeoyl-CoA to produce compounds with an ortho-diol on one of the two aromatic rings, such as 3^′^-hydroxy-bisdemethoxycurcumin (**A**) and 3^′^-hydroxy-demethoxycurcumin (**B**) (see [6] Figure 1). To further investigate the role of PKSs in ginger/turmeric specialized metabolism, we identified >40 unitrans as putative PKSs by comparing representative type III PKS genes [30] against the ArREST database using BLASTX. These potential PKS genes belong to three major groups: chalcone/naringenin-chalcone synthases (CHSs), relatives of a polyketide synthase from *Wachendorfia thyrsiflora* (Haemodoraceae) (WtPKS1) and hydroxycinnamoyl-diketide synthase (DKS) from turmeric [31-34], and relatives of curcuminoid synthase (CURS) from turmeric [33,34], as well as a diverse group of putative polyketide synthases (Figure 2, Additional file 1: Table S5). The ginger and turmeric enzymes tentatively identified as CHS or naringenin-CHS showed much lower expression levels compared to the other two classes in turmeric, based on EST number. CHS ESTs were not detected in GW and were only found in the GY rhizome. Ginger is not known to produce large amounts of flavonoids, which would be produced by these CHS enzymes, and instead is known for gingerols, gingerol-related compounds and a diversity of diarylheptanoids. Indeed, our metabolic profiling work with these ginger lines [22] suggests that flavonoids are only minor constituents of these plants, even though the phenylpropanoid pathway appears to be very active (see above). Thus, a combination of low expression of CHS and competition by other PKS-like enzymes may deplete substrate pools for CHS, thus preventing ginger from accumulating flavonoids to appreciable levels.

In contrast, members of the ginger/turmeric WtPKS1/DKS and CURS-like subclasses in aggregate are ubiquitously expressed in the rhizome, leaf and root tissues of both ginger and turmeric. The namesake protein of the first class, WtPKS1 is the first type III PKS reported in the biosynthesis of diarylheptanoids [35] and is neither a curcuminoid or gingerol synthase, but is rather a diketide synthase. Other members of this sub-family have been shown to belong to the curcumin synthase-like subclass. Certain members from both groups of enzymes are involved, apparently, in production of curcumin *in planta*; they have been demonstrated to produce curcumin in vitro when expressed in recombinant form. None of these genes have been shown to be involved in the production of gingerols. Another gene of this class (Os07g17010) was identified in rice and also annotated as a “curcuminoid synthase” despite the fact that rice does not produce curcuminoids [31]. Expressed exclusively at low levels in the developing rice anther, this protein likely plays a different role *in vivo* than what is suggested from *in vitro* analysis of the recombinant rice protein. Moreover, Os07g17010 is not closely related to WtPKS1 or to any of curcuminoid synthase or diketide synthase genes identified from curcuminoid-rich ginger and turmeric (see Figure 2). As a result, we cannot determine the exact roles of all of the members of the class of WtPKS1 gene family in ginger and turmeric at this time. Nevertheless, available data so far suggest that the large array of PKS-derived compounds in ginger and turmeric may be the result of multiple PKS-like enzymes catalyzing slightly different reactions, each with different substrate specificities and product outcomes. Other ginger and turmeric genes from the WtPKS1/curcumin synthase-like subclass are excellent candidates for involvement in these processes, and are the subject of ongoing investigation. Furthermore, the β-ketoacyl-CoA synthase-like subclass (Figure 2) is also noticeably expanded in ginger and turmeric relative to other species and may also play a role in production of these compounds.

Other enzymes required to decorate or modify the diarylheptanoid and gingerol-related backbone structures could well belong to the other major gene families evaluated for expression. For example, specific reductases and hydroxylases are likely to be involved in elimination of double bonds and in forming hydroxyl groups on the compounds found in these plants, and these classes of enzymes were well represented in the ArREST database. Many of these potential genes show relatively high expression in all or most tissue types of ginger/turmeric (see Additional file 1: Table S5). These results provide important clues for further research to elucidate the pathways/ networks involved in producing diarylheptanoids and gingerol-related compounds in these plants.

### Terpenoid biosynthesis in ginger and turmeric

Terpenoids are another major class of bioactive compounds found in ginger and turmeric [22,25,36-40]. Isopentenyl diphosphate and dimethylallyl diphosphate (IPP and DMAPP), the common building blocks for mono-, sesqui- and other terpenoids, appear to be produced mainly by the plastidic methylerythritol phosphate (MEP) pathway in these species as ESTs for all enzymes in this pathway were readily identified in the ArREST database at high levels (Additional file 1: Table S4), especially the potential regulatory enzyme, 1-deoxy-D-xylulose-5-phosphate (DOXP) synthase. In contrast, two important enzymes of the cytosolic mevalonate (MVA) pathway, phosphomevalonate kinase and pyrophosphomevalonate decarboxylase, were not detected in the database. Also, other genes in the MVA pathway were represented by very low EST levels for all tissues, even those producing high levels of sesquiterpenoids, which are derived from farnesyl diphosphate, a compound believed to be synthesized in the cytosol of most plants. These results suggest that the MEP pathway is the essential pathway for production of precursor IPP/DMAPP involved in the biosynthesis of terpenoids found at high levels in ginger and turmeric, including sesquiterpenoids. Furthermore, the transport of IPP/DMAPP out of the plastid to the cytosol is likely to also occur in these plants, as has been shown for other plants such as snapdragon and sweet basil [19,41].

**Figure 3:**
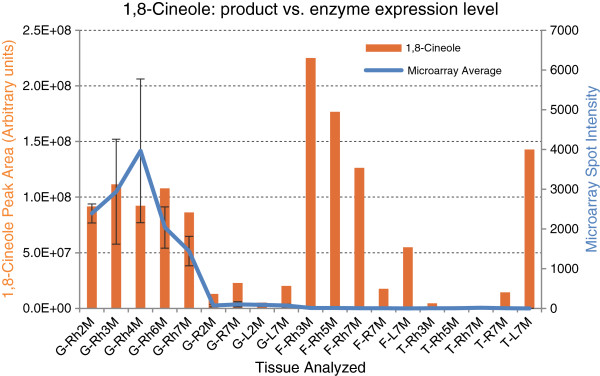
**Comparison of GC/MS-determined 1,8-cineole levels to cineole synthase gene expression profiles generated by DNA microarray analysis.** 1,8-Cineole levels in either turmeric or ginger are shown as bar graphs. Gene expression levels from normalized microarray spot intensities are shown as the line graph. Error bars indicate standard errors of the mean. The sequence for 1,8-cineole synthase used in this comparison was identified from ginger rhizomes. Both metabolite profiling and gene expression profiling of the ginger 1,8-cineole synthase match very well for ginger. However, 1,8-cineole produced in turmeric appears to be produced by an enzyme that is not the same as that from ginger. Bottom axis abbreviations represent developmental time courses for three cultivars, G: white ginger, F: turmeric variety FMO (Fat Mild Orange), and T: turmeric variety TYA (Thin Yellow Aromatic), respectively. Rh: rhizome, R: root, L: leaf. 2 M, 3 M, 4 M, 5 M, 7 M represent plants of different ages in months, e.g., 2 M: 2 months, 3 M: 3 months, etc.

Only two terpene synthases (TPS), a germacrene D synthase and (*S*)-β-bisabolene synthase from ginger, have been reported from either of these species [11,42]. However, the ArREST database contains 45 unitrans identified as putative TPSs including: 19 monoterpene synthases, 11 sesquiterpene synthases, 2 diterpene synthases, 3 triterpene synthases and 10 tetraterpene synthases (Additional file 1: Table S5). Most of the TPS unitrans in the ArREST database possess few ESTs (average 2.47), several may represent different regions of the same gene (such as 5^′^ and 3^′^ regions), and all putative triterpene synthases appear to be exclusive to the rhizomes. Two of the unitrans appeared to represent full-length monoterpene synthase (MTS) cDNAs, one from ginger rhizome and the other from turmeric leaf. The corresponding recombinant protein from ginger rhizome was expressed in *E. coli* and assayed for enzymatic activity. The ginger MTS catalyzed the formation of 1,8-cineole and small amounts of *p*-menth-1-en-8-ol, which is a intermediate product during GPP conversion to 1,8-cineole. Although all ginger tissues produce 1,8-cineole, the rhizome contains much more than root or leaf tissues. Gene expression profiling from a microarray analysis also verify that 1,8-cineole synthase is predominantly expressed in the rhizome (Figure 3). Although the turmeric rhizome also produces large amounts of 1,8-cineole, this ginger MTS was not expressed in the turmeric rhizome according to the microarray analysis, suggesting that there is/are other 1,8-cineole synthase(s) in the turmeric rhizome.

In addition to the TPSs discussed above, other gene families possibly involved in ginger and turmeric terpenoid biosynthesis are easily identified in the database, including P450s. Of these, a limonene hydroxylase-like enzyme (CYP71D class) is one of the most highly expressed P450s (see Additional file 1: Table S6), based on EST counts. This enzyme class is associated with the biosynthesis of oxygenated monoterpenoids (i.e. carvone, menthone, menthol, and pulegone, etc.). Because these specific compounds have not been detected in ginger or turmeric, CYP71D in ginger and turmeric plants is likely to be involved in producing highly accumulating compounds such as the turmerones, suggesting that this enzyme class may have evolved unique functions in these two plants.

### Conservation of rhizome-enriched genes

In an attempt to discover genes involved in defining rhizome tissue identity and rhizome development, we compared 1,223 rhizome-specific ESTs from *Sorghum* (see Additional file 3) [12] to our ArREST database using TBLASTX. As a result, 2,383 ginger/turmeric unitrans containing 8,017 ESTs were identified as having significant homology (E ≤ 1 × 10^-10^) to the *Sorghum* rhizome ESTs. Of these, 1,606 ginger/turmeric unitrans (6,425 ESTs) were expressed in tissues besides the rhizome, leaving 777 unitrans (1,592 ESTs) that appeared to be exclusively expressed in ginger or turmeric rhizome (according to EST data). Within this group of 777 rhizome-enriched unitrans, 70.6% or 547 unitrans (1,124 ESTs) had GO annotations in “biological process”, compared to 87.6% of the entire ArREST database. The remaining unknown unitrans, while lacking any known biological function, appear to represent actual genes and not random or “junk” sequence data. This result corresponds to earlier findings for Johnsongrass rhizomes [12,43].

**Figure 4:**
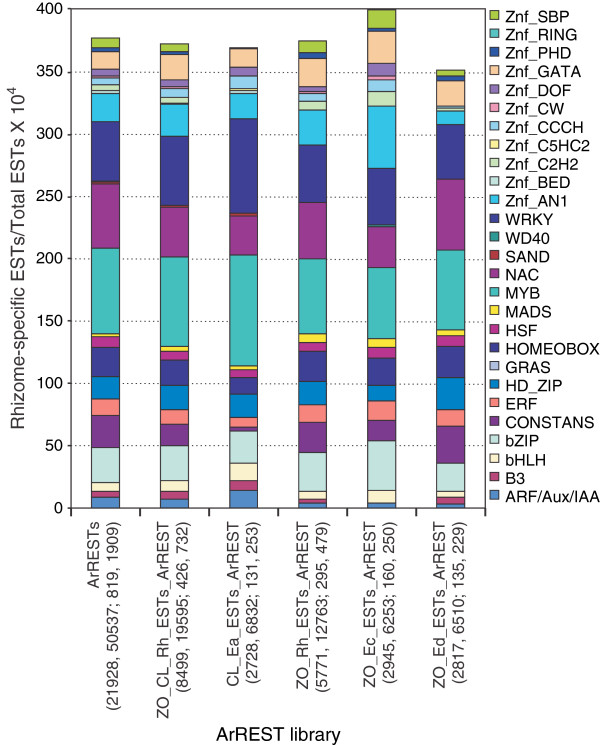
**Fraction of ESTs (standardized per 10,000 ESTs) of different classes of transcriptional regulators (GO category 0003677) in ginger and turmeric libraries.** Library descriptions are listed under the graph, followed by additional information per library in the parenthesis: (number of unitrans, number of ESTs, number of unitrans with GO:0003677, number of ESTs with GO:0003677). ArRESTs: EST collection of all ginger and turmeric libraries within ArREST. ZO_CL_Rh_ESTs_ArREST: combined ESTs of all ginger and turmeric rhizome libraries within ArREST. CL_Ea_ESTs_ArREST: turmeric rhizome library. ZO_Rh_ESTs_ArREST: combined EST collection of two ginger rhizome libraries. ZO_Ec_ArREST: White ginger rhizome EST library. ZO_Ed_ArREST: Yellow ginger rhizome library. Values per category are shown in Additional file 1: Table S8.

The rhizome-enriched ESTs were enriched (2-fold more compared to leaf or root) for genes involved with “protein modification” (GO:0006464; Additional files 1 and 2: Table S7 and Figure S5), with 50% of these possessing homology to genes associated with kinase-mediated signal transduction, and the remainder having homology to other serine-threonine kinases or ubiquitin-associated activities. Such post-translational protein modifications are suggestive of possible roles in biotic/abiotic stress response and phytohormone signal transduction [44-47]. In contrast, a number of GO categories were noticeably deficient. For example, few unitrans were found with GOs associated with transport or cell organization and biogenesis (Figure 4) (these were primarily devoted to cell wall biosynthesis and lignification [48,49]), while 37% of the ESTs in the “catabolism” gene ontology are actually involved with the early stages of the phenylpropanoid pathway [50,51]. Other GO categories underrepresented in the rhizome include nucleotide/nucleic acid metabolism and generation of precursor metabolites/energy (Additional file 2: Figure S5). This apparent lack of a diversity of metabolic processes displays a bias in the rhizome toward processes associated with cell wall biosynthesis and remodeling as well as specific specialized metabolic pathways.

### Identification of transcriptional regulators in ginger and turmeric rhizomes

MYB factors easily dominated other classes of transcription factors in both unitrans and EST numbers in the total ArREST database (Figure 4, Additional file 1: Table S8). The other major groups of transcription factors are the NAC, WRKY, homeobox, bZIP and CONSTANS classes. This contrasts with what has been found in either *Arabidopsis thaliana* or *Oryza sativa*, where the basic helix-loop-helix (bHLH) family is one of the largest families of transcription factors, closely followed in number by the MYB proteins [52-55]. In the case of ginger and turmeric, this trend is reversed: the MYB class is the most highly expressed class of transcription factors and apparently possesses the largest number of distinct genes, whereas the bHLH class is one of the lowest abundant classes (see Figure 4, Additional file 1: Table S8), based on EST counts and confirmed by additional expression profiling (see below). Although this trend may merely be a reflection of the genes being transcribed rather than the actual genomic content, it is an interesting finding. These two classes of transcription factors have been shown to complex together and regulate a variety of processes, most notably the specification of hairy trichomes, root hairs and petal conical cells in Arabidopsis and *Antirrhinum majus*[56-59]. In addition, both ginger and turmeric possess noticeably expanded numbers of the WRKY and NAC types of transcription factors compared to Arabidopsis and rice. Both WRKY and NAC transcription factors have also been shown to play integral roles in plant defense, stress response and development [60-63].

In the case of ginger, turmeric and *Sorghum* rhizomes, it will be interesting to see which genes are regulated by these classes of transcription factors, because rhizomes lack trichomes and root hairs and do not typically accumulate appreciable levels of anthocyanins. Anthocyanin production and trichome development in plants are processes known to be regulated by MYB proteins [64,65]. A plausible role for MYB proteins in ginger and turmeric rhizomes might lie in the regulation of rhizome-specialized metabolism (particularly the phenylpropanoid-derived diarylheptanoids or gingerols in ginger and turmeric) or general rhizome structure and development.

**Figure 5:**
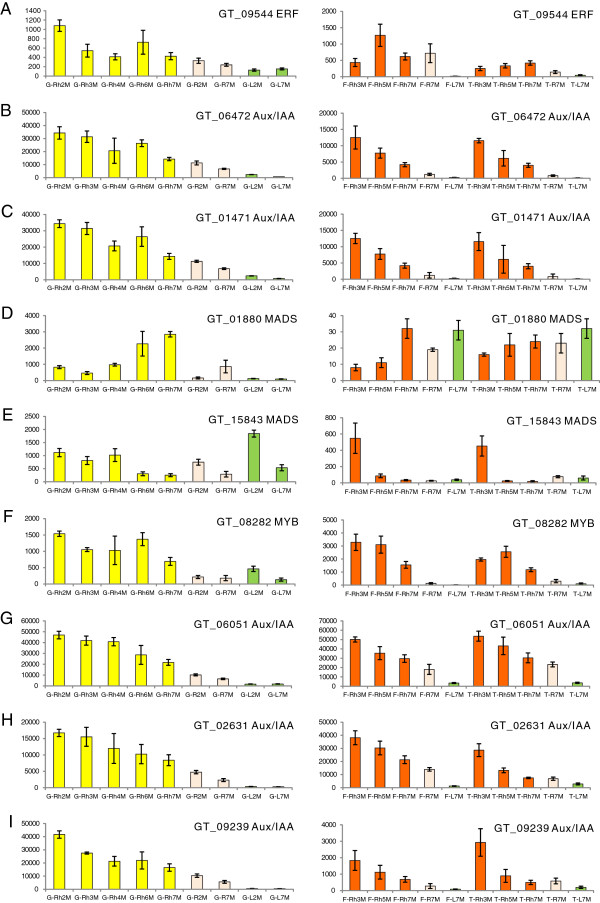
**Comparisons of microarray signal intensities of several rhizome-enriched transcription factor unitrans in ginger and turmeric.** Each of these selected genes possesses signal intensities with rhizome–enriched expression in at least one of the species compared to other tissues and/or time-points with coefficients ≥ 2 and p-values ≤ 0.05.

To verify the rhizome-enriched expression of specific transcriptional regulators, we analyzed the expression patterns of 745 of the 777 rhizome-enriched data set using a custom oligonucleotide-based microarray (the other 32 genes did not yield good quality oligos for inclusion in the array). Ten unitrans putatively encoding transcriptional regulators were expressed at higher levels (expression coefficients >2 and p-values ≤0.05) in rhizome versus other tissues in various tissue and/or age specific comparisons in both ginger and turmeric: a MYB, an ethylene response factor (ERF), 2 MADs, 3 auxin response factors (ARFs) and 2 AUX/IAA transcriptional regulators (Figure 5). It is notable that 7 of the 10 transcriptional regulators identified in this manner (the ERF and 5 AUX/IAAs, Figure 5) appear to encode phytohormone-related proteins and are significantly up-regulated in the rhizome tissues at several time points in both ginger and turmeric. Two other genes shown by the microarray experiments to be up-regulated within the rhizome of ginger or turmeric, respectively, were the MADS box genes GT_01880 and GT_15843 (Figure 5D & E). Whether these two genes play complementary or different roles in these two species remains unclear. Nevertheless, these results suggest roles for auxin and ethylene in the establishment or maintenance of rhizome cell fate or rhizome apical dominance [66-68].

### Identification of genes involved in rhizome development

AUX/IAA proteins have been implicated in the development of auxin-dependent vascular tissues [69]. The presence of AUX/IAA proteins with rhizome-enriched expression is notable because auxin has been proposed to repress the initiation of shoots from rhizomes in other rhizomatous species [15,16]. The simplest explanation for the role of these transient proteins is that the AUX/IAA ESTs observed represent the basal transcripts produced in the rhizomes. The corresponding translated AUX/IAA proteins would bind to and inhibit their ARF counterparts that otherwise directly control gene expression via DNA binding [70,71]. However, since auxin from the shoot is readily available in the rhizome, the AUX/IAA proteins would be quickly degraded by a complex analogous to the auxin responsive SCF complex [72], allowing for ARF-DNA binding. As a result, the ARF would not play the role of a transcriptional activator, but rather of a transcriptional repressor. As repressors, these ARF proteins would bind to their respective promoter regions and repress shoot development, as well as possible transcription of relevant ARF genes. This would help explain the lack of putative ARF genes in the 777 ESTs common to rhizomes from ginger/turmeric and *Sorghum*.

Although apical dominance is pronounced in rhizomes of *S. halepense*[73], it appears to be reduced in ginger and turmeric, possibly due to the differential presence/absence of specific NAC transcription factors. Such a hypothesis is plausible because the rhizome is a stem and mutations in NAC transcription factors have been associated with loss of apical dominance in stems [74]. NAC proteins, which may also regulate various aspects of meristematic development like rhizome bud dormancy, are known to be expressed in monocot meristems [75] and to be regulated by auxin via a similar auxin-responsive ubiquitination process [76,77].

Ethylene was also implicated in the maintenance of the rhizome as a distinct tissue by the abundance of ESTs in the rhizomes of ginger, turmeric, and *Sorghum* for genes associated with ethylene signaling (ERF proteins); the potential role of this phytohormone in rhizome biology has also been suggested for other species [17,18]. Ethylene may play a role in both the promotion of rhizome elongation and the suppression of shoot development. Could shoot-derived auxin be stimulating ethylene evolution in the rhizome, thereby repressing shoot formation? This idea has been hinted at in earlier experiments where the addition of auxin resulted in increased production of ethylene from exposed plant tissues [78,79], although rhizomes were not tested. However, this hypothesis does not completely explain the previously observed roles of gibberellins in rhizome growth and development [18,80]. A possible explanation is that gibberellins may be acting as agents in the crosstalk between auxin stimulus and the ethylene response pathways. Such a relationship has been suggested for other tissues such as stem, root, and tuber [81-83], but has not been established for rhizomes.

**Figure 6:**
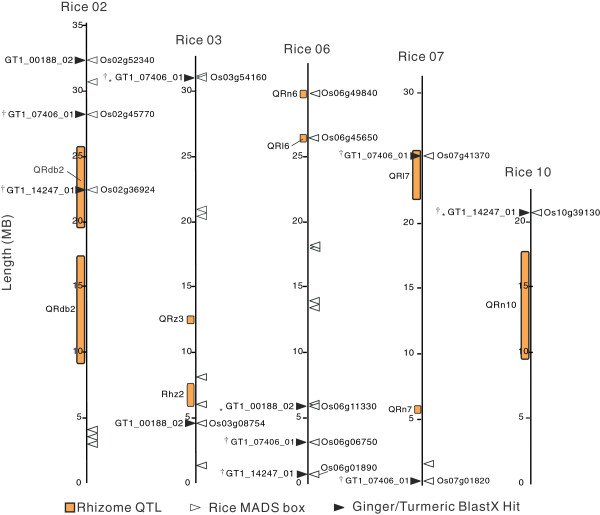
**Associations between MADS box transcription factors from ginger and turmeric, sorghum rhizome-enriched ESTs, and rice rhizomatous QTLs on rice chromosomes 2, 3, 6, 7, and 10.** Orange boxes represent rhizome QTLs, while triangles indicate the spatial locations of MADS box genes on the rice chromosomes. Rice MADS box genes are illustrated using white triangles; black triangles are ginger/turmeric Blastx hits (e-values < e^-20^). * denotes the best Blastx hits for the ginger and turmeric contig/rice gene comparison. † denotes a MADS box gene shown by microarray analysis to be preferentially expressed in the rhizome when compared to leaf or root tissues (Figure 3D & E).

MADS box transcription factors may also play an important role in rhizome initiation and development. Three (GT_01880, GT_13200, and GT_15843) of the eight MADS box unitrans that were identified in rhizomes appeared to be expressed exclusively in the rhizomes of ginger, turmeric and *Sorghum*, based on EST data. Microarray analysis confirmed rhizome-specific expression for one of these genes GT_01880 (Figure 5D). This gene appears to be homologous to MADS box genes whose positions are close to quantitative trait loci (QTLs) (Figure 6) associated with rhizomatousness in *Oryza* and *Sorghum*[12,43]. In addition, related rice MADS box proteins have been implicated as possibly having roles in flower development; flower tissue was not included in our analysis due to the difficulty in obtaining this tissue from these plants. Possible functional overlap between flower and rhizome development is not unreasonable and some MADS box transcription factors have been implicated in the development of both floral and vegetative tissues such as tubers and rhizomes [13,14]. It will be very interesting to determine if the proteins encoded by these three unitrans do in fact play some role in controlling or directing rhizomatous growth and development.

## Conclusion

We have analyzed ESTs from two ginger lines (white ginger and yellow ginger) and one turmeric line (orange turmeric) and investigated the expression of the corresponding genes in rhizome, root and leaf tissues. Many candidate genes for involvement in many core and specialized metabolite pathways were present and highly expressed in especially the rhizomes of these plants. Several transcription factors and transcriptional regulators were specifically expressed in ginger and turmeric rhizome, with corresponding homologs expressed in rhizome of Johnsongrass, a rhizomatous, invasive grass. These rhizome specific transcription factors may be involved in regulation of rhizome growth and development.

## Methods

### cDNA library construction and sequencing

Using the white ginger (GW), yellow ginger (GY), and red/orange turmeric (T3C) lines described previously [2,5], total RNA was extracted from rhizomes, young leaves, and roots using the method of Dong and Dunstan [84]. Poly(A)^+^ RNA was purified from 1000 μg of total RNA using the PolyATract® mRNA isolation kit (Promega, USA) and cDNA was synthesized from 1 μg of poly(A)^+^ RNA using a Uni-ZAP ® XR cDNA synthesis kit (Stratagene, USA) according to the manufacturers’ instructions. The directionally cloned (*Eco*RI/*Xho*I) cDNA libraries were then mass-excised *in vivo* and the resulting phagemids (pBluescript SK(−)) were propagated in the *E. coli* strain TJC-121 [85]. Individual cDNA clones containing inserts were sequenced from the 5′and 3′ ends using the T7 and T3 promoter sequencing primers, respectively.

### Production of the EST database in PAVE

ESTs were assembled with the Program for Assembling and Viewing of ESTs, PAVE [86].

### Classifying unitrans by gene ontology

All unitrans within the ArREST database were assigned UniProt IDs based on BLASTX results. Microsoft Access was used to assign GO terms from the Gene Ontology Annotation (GOA) Database file, gene_association.goa_uniprot.gz (http://www.ebi.ac.uk/GOA/index.html) to ginger and turmeric EST unitrans based on their corresponding UniProt IDs. Of the remaining 5,587 unitrans lacking GOs, 1,179 had GOs using the GO annotation search tool (http://www.arabidopsis.org/tools/bulk/go/index.jsp). GO annotations were assigned to additional 327 unitrans via the ArREST-PAVE website. The remaining 4,081 unitrans that completely lacked GO annotations were compared again against both the Swiss-Prot and TrEMBL databases separately using BLASTX (E-value ≤ 1E-10). We examined the 5 best hits from both BLASTX results for each unitrans and, if necessary, considered all remaining hits until we found a hit possessing a GO annotation. This approach allowed us to annotate 87.6% of our ginger/turmeric unitrans, leaving 12.4% of the unitrans unannotated. The unitrans were assigned to their appropriate gene ontology categories using the map2slim program and the goslim_plant.obo file downloaded from the Gene Ontology website (http://www.geneontology.org/GO.slims.shtml).

### Rhizome-enriched transcripts from ginger, turmeric and sorghum

To determine rhizome-enriched transcripts common to ginger/turmeric and *Sorghum* species, a subtractive-reciprocal best BLAST hit approach was used [87] followed by direct TBLASTX (E-value ≤ 1E-10) comparisons of the species- and tissue-specific libraries (turmeric leaf vs. turmeric rhizome; combined ginger leaf vs. ginger rhizome; and combined turmeric leaf/rhizome vs. combined ginger leaf/root/rhizome). Nonredundant unitrans sequences were sorted using Microsoft Access into three categories: unique to rhizome, other tissue, and shared in all tissues. Unitrans exclusive to ginger or turmeric rhizome were used in a TBLASTX (E-value ≤ 1E-10) comparison with 1,223 publicly available *Sorghum* rhizome EST sequences (see Supplementary Information, http://www.ncbi.nlm.nih.gov/) from either *S. propinquum* or *S. halepense*[12]. The reciprocal best BLAST hit approach was used, but in contrast to the comparisons in and between ginger and/or turmeric, the reciprocal best hits produced in this assessment were considered to contain possible orthologs required for rhizomatous tissue identity and/or function.

### Identification and evaluation of probable transcription factors and transcriptional regulators in ginger and turmeric

In order to identify possible *trans*-acting transcriptional regulators and transcription factors within the ArREST database, we queried the unitrans library for sequences with the associated gene ontology identifier for DNA binding: GO0003677. These queries produced 1,372 nonredundant unitrans with this particular gene ontology identification, which were then analyzed using the protein motif identification program INTERPROSCAN [88] to identify any possible non-generalized DNA binding domains. Following analysis with INTERPROSCAN, the 1,372 unitrans were manually curated to purge unitrans possibly associated with generalized transcriptional machinery, yielding a total of 818 unitrans that were then tallied to determine the number of unitrans or ESTs belonging to each of the DNA binding domain categories.

### Mapping of putative ginger/turmeric mads-box transcription factors to rice

To determine if the ginger/turmeric MADS-box transcription factors corresponded to known QTLs associated with rhizomatousness [43], 3 rhizome-enriched ginger/turmeric unitrans identified as having significant homology to ESTs found in *Sorghum* rhizomes [12] were compared to the IGRSP build 4.0 pseudomolecules/annotations (International Rice Genome Sequencing Project 2005). A number of rice genes were identified as possible orthologs of the ginger/turmeric unitrans. The annotations of these genes were retrieved using the various search tools available on Gramene [89]. Furthermore, annotations for all predicted rice MADS-box proteins, QTLs and their associated simple sequence repeat (SSR) primer pairs were also retrieved using Gramene [12,43,90,91]. These annotations were converted manually into a general feature format (GFF) file and loaded into the Apollo genome editor [92], along with the appropriate IGRSP build 4.0 pseudomolecule (International Rice Genome Sequencing Project 2005). As a result, a number of virtual maps of rhizomatousness QTLs and their probable spatial relationships to the positions of ginger/turmeric/rice MADS box transcription factors on the IGRSP pseudomolecule were produced.

### Microarray analysis of ginger and turmeric genes

A custom microarray was produced by Agilent using oligos designed by us from the ArREST database. This array and its design are available through Agilent’s eArray site (https://earray.chem.agilent.com/earray/) as published design Z.Officinale/C.Longa. Oligo design procedures, experimental design and experimental steps are outlined in Additional file 4. Spot intensities were extracted from the scanned microarray images using Agilent Feature Extraction software, and data analysis was performed using R [93], Bioconductor (PUBMED: 16939789), and limma [94,95]. Normalization within and between arrays was carried out using the limma normalizeWithinArrays and normalizeBetweenArrays functions, utilizing the loess method [96] for within array normalization and the quantile method for between array normalization. A linear model containing each of the sample types (as defined by the combination of turmeric or ginger cultivar, time of harvest, and tissue), plus a term to account for differences in intensity due to the labeling fluorochrome (Cy3 vs. Cy5), was then applied to the data using the limma lmFit function. The contrasts of interest were calculated using the contrasts.fit function and their significance (statistical analysis) was determined using the eBayes function in limma. The resulting p-values were adjusted for multiple comparisons using the write.fit function employing the Benjamini-Hochbergfalse-discovery rate adjustment [97].

### Cloning, expression and enzyme assay of 1,8-cineole synthase

PCR product amplified with 5^′^-ATGAGGAGGTCGGGAAATTACCA-3^′^ and 5^′^-GAGCTGGACAGGCTCGATCA-3^′^ using Pfu polymerase was inserted into the pCRT7CT-TOPO vector (Invitrogen), which was transformed into BL21 (DE3) CodonPlus RIL (Stratagene) and expressed for 18 h at 18°C with 0.005 - 0.4 mM of IPTG. After induction, the pellet of E. coli was vortexed with Washing Buffer (20 mM Tris–HCl, pH 7.0, 50 mM KCl) and then centrifuged. Protein Extraction Buffer (50 mM 3-(N-morpholino)-2-hydroxypropanesulfonic acid, pH 7.0, 10% [v/v] glycerol, 5 mM MgCl_2_, 5 mM DTT, 5 mM sodium ascorbate, 0.5 mM phenylmethylsulfonyl fluoride) was added to washed E. coli pellet and vortexed, sonicated and centrifuged. Supernatant was recovered and the buffer was changed to Enzme Assay Buffer (10 mM 3-(N-morpholino)-2-hydroxypropanesulfonic acid, pH 7.0, 10% [v/v] glycerol, 1 mM DTT) using PD-10 column (GE Healthcare Life Sciences). Divalent cations (20 mM MgCl_2_, 0.5 mM MnCl_2_ at final concentration), protease inhibitors (0.2 mM NaWO_4_, 0.1 mM NaF at final concentration) and either geranyl diphosphate (GPP, 10 μg) or farnesyl diphosphate (FPP, 10 μg) were added to total 500 μl of Enzyme Assay Buffer containing soluble proteins and incubated for 3 h at 30°C with 200 μl of top-layered pentane. Either top pentane and/or vortexed, centrifuged pentane was used for metabolite analysis on a Rtx-5MS w/ 5 m Integra-Guard Column (Restek, 0.25 mm ID, 0.25 μm df, 30 m) in a Thermo Finnigan Trace GC 2000 coupled to a DSQ mass spectrometer. Product identification was performed by comparison of retention time and mass spectra to known standards.

The data sets supporting the results of this article are available in the NCBI dbEST (Database of Expressed Sequence Tags) repository under accession nos. DY344695 – DY395309, beginning with http://www.ncbi.nlm.nih.gov/nucest/DY344695.

## Competing interests

The authors declare that they have no competing interests.

## Authors’ contributions

HJK, XM and JK performed experiments. HJK, ETM, XM, ZX, KG and DRG analyzed data. XM, HJK, ETM, JK and DRG wrote the manuscript. DRG designed experiments. HRK, YY, DK and RAW performed EST sequencing. CAS constructed PAVE. All authors helped edit the manuscript or reviewed the manuscript. All authors read and approved the final manuscript.

## Supplementary Material

Additional file 1: Table S1 cDNA library sources for sequences and unigene sets described in this study. **Table S2.** The most abundantly represented transcripts in the ArREST database (EST number ≥ 40). **Table S3.** The most abundantly represented transcripts in specific ginger and turmeric rhizome libraries (EST number ≥ 10 per library). **Table S4.** Normalized EST expression levels for selected enzymes in ginger and turmeric metabolic pathways. **Table S5.** Normalized EST expression levels for selected gene families. **Table S6.** Normalized EST expression levels for cytochrome P450 monooxygenases. **Table S7.** Normalized percentage of ArREST ESTs with GO associations. **Table S8.** Probable transcriptional regulator classes within ArREST associated with GO:0003677. Click here for file

Additional file 2: Figure S1 Distribution of EST sequence length. The average EST sequence length was 817 bp, with unitrans (unique transcripts, a.k.a. contigs) lengths ranging from 151 to 4021 bp, with the greatest number of unitrans having between 701 and 800 bp, and with 95% exceeding 300 bp. **Figure S2.** Gene Ontology (GO) annotations of ArREST unitrans. 87.6% of the ArREST unitrans could be annotated by GO classification. Eight GO categories had EST abundances greater than 5%, including protein modification (10.5%), transport (9.2%), metabolism (9.0%), transcription (8.6%), cellular process (8.0%), protein biosynthesis (6.9%), electron transport (5.6%), and biological process unknown (5.3%). In contrast, the GO category secondary metabolism contained relatively few ESTs (0.3%). **Figure S3.** Proposed metabolic map showing how the curcuminoids, gingerols and terpenoids are produced from sucrose in a large interconnected network. Names of enzymes identified in the ArREST database are colored blue; % values indicate fraction of unitrans in the database that are represented by genes encoding each protein. **Figure S4.** Phylogenetic tree of P450 monooxygenases. A neighbor joining tree was generated with 1136 P450s including 170 ginger and turmeric P450s from the ArREST database (indicated by black diamonds), 247 Arabidopsis P450s, 350 rice P450s and 369 P450s from other plants. P450 subfamily classifications are indicated. **Additional file**1**: Table S6** contains a summary of these data for ginger and turmeric. As the tree is so large, it was impossible to display it at readable scale on a page size that is typical. Thus, details of tree can been seen by zooming in on this page of the PDF file. **Figure S5.** Overall gene expression in rhizomes is similar to but distinct from that observed for other plant tissues. Total ArREST ESTs, ESTs with shared expression in ginger or turmeric rhizomes and at least one other ginger or turmeric tissue, and ESTs found exclusively in ginger or turmeric rhizomes are represented as light grey, dark grey and black bars, respectively. Values used to generate this graph are presented in **Additional file**1** :Table S7.** Click here for file

Additional file 3 GenBank Accessions for Rhizome-specific Transcripts from Sorghum. Click here for file

Additional file 4 Microarray experimental methods, oligonucleotide probe design, microarray design (interwoven design). Click here for file

## Financial source

U.S. National Science Foundation Plant Genome Program Grant #DBI-0227618.
