# Supplementary material for: Ginger and turmeric expressed sequence tags identify signature genes for rhizome identity and development and the biosynthesis of curcuminoids, gingerols and terpenoids
Source: BMC Plant Biol. 2013 Feb 15;13:27. doi: 10.1186/1471-2229-13-27 (PMC3608961; doi:10.1186/1471-2229-13-27)
Supplement: Additional file 1: Table S1 — cDNA library sources for sequences and unigene sets described in this study. Table S2. The most abundantly represented transcripts in the ArREST database (EST number ≥ 40). Table S3. The most abundantly represented transcripts in specific ginger and turmeric rhizome libraries (EST number ≥ 10 per library). Table S4. Normalized EST expression levels for selected enzymes in ginger and turmeric metabolic pathways. Table S5. Normalized EST expression levels for selected gene families. Table S6. Normalized EST expression levels for cytochrome P450 monooxygenases. Table S7. Normalized percentage of ArREST ESTs with GO associations. Table S8. Probable transcriptional regulator classes within ArREST associated with GO:0003677. [file 1471-2229-13-27-S1.pdf]

Supplemental Table S1. cDNA library sources for sequences and unigene sets described in this study

Species	Line	Tissue	LibID	ESTs	Contigs*	Unique Contigs**	Singletons	Unitrans***
Ginger	GY	Rhizome	ZO__Ed	5653	2280	1145	337	2617
		Leaf	ZO__Ea	5989	2034	907	193	2227
		Root	ZO__Ee	5640	2241	1053	301	2542
	GW	Rhizome	ZO__Ec	6226	2500	1202	400	2900
		Leaf	ZO__Eg	7108	2570	1302	133	2703
		Root	ZO__Ef	6283	2576	1342	329	2905
Turmeric	T3C	Rhizome	CL__Ea	6471	2385	1870	301	2686
	(FMO)	Leaf	CL__Eb	6769	2342	1692	293	2635
Total				50139	18928	10513	2287	21215

*Number of contigs that contain at least one EST from the library
**Number of contigs that contain ONLY ESTs from the library
*** Combined counts for Contigs and Singletons per library

Supplemental Table S2. The most abundantly represented transcripts in the ArREST database (EST number ≥ 40)*

Unitrans ID	# of ESTs Matching Top UniProt Hit	Top UniProt E-value	Description of Top UniProt Hit with Known Function
GT_00001	124	0	Rubisco activase (*Zantedeschia aethiopica*, RCA4)
GT_00003	116	4.00E-82	Ribulose bisphosphate carboxylase small chain, chloroplastic (*Musa acuminata*, RBCS1)
GT_00002	97	8.00E-20	Metallothionein-like protein (*Elaeis guineensis* var. *tenera*, MT-3)
GT_00004	91	9.00E-73	Ribulose bisphosphate carboxylase small chain, chloroplastic (*Musa acuminata*, RBCS1)
GT_00005	83	0	Catalase (*Musa acuminata*, Cat2)
GT_00006	83	0	S-adenosylmethionine synthetase (*Dendrobium crumenatum*, SAMS)
GT_00007	82	7.00E-82	Ribulose bisphosphate carboxylase small chain, chloroplastic (*Musa acuminata*, RBCS1)
GT_00008	78	0	Rubisco activase (*Zantedeschia aethiopica*, RCA4)
GT_00009	66	2.00E-135	Protein disulfide-isomerase (*Ricinus communis*, PDI)
GT_00010	66	0	Heat shock cognate 70 kDa protein, putative, expressed (*Oryza sativa* subsp. *japonica*, HSP72)
GT_00011	63	0	Ubiquitin (*Pisum sativum*, PUB3)
GT_00012	59	1.00E-39	Photosystem II 10 kDa polypeptide, chloroplastic (*Nicotiana tabacum*, PSBR)
GT_00013	59	0	Rubisco activase (*Zantedeschia aethiopica*, rca4)
GT_00014	57	1.00E-49	4-Coumarate--CoA ligase-like 1 (*Oryza sativa* subsp. *japonica*, 4CLL1)
GT_00015	54	2.00E-130	Chlorophyll a/b binding protein CP29 (*Phaseolus aureus*, CP29)
GT_00016	53	6.00E-118	Light-harvesting complex I protein (*Populus trichocarpa*, LHCA4)
GT_00017	52		No match
GT_00018	51	3.00E-139	Chlorophyll A-B binding protein (CAB), putative (*Musa acuminata*, CAB)
GT_00019	50	0	Glyceraldehyde-3-phosphate dehydrogenase B, chloroplastic (*Pisum sativum*, G3BP)
GT_00020	47	0	Fructose-bisphosphate aldolase, chloroplastic (*Oryza sativa* subsp. *japonica*, ALFC)
GT_00021	45	1.00E-108	Chlorophyll a-b binding protein 6A, chloroplastic (*Solanum lycopersicum*, CB11)
GT_00022	45	9.00E-142	Chlorophyll a-b binding protein 36, chloroplastic (*Nicotiana tabacum*, CAB36)
GT_00024	42	0	Polyubiquitin (*Sporobolus stapfianus*, UBIQ)
GT_00025	42	3.00E-48	4-coumarate--CoA ligase-like 1 (*Oryza sativa* subsp. *japonica*, 4CLL1)
GT_00026	41	3.00E-137	Endochitinase A2 (*Zea mays*, CHI)
GT_00027	41	0	Polyubiquitin (*Zea mays*, UBIQ)
GT_00028	41	9.00E-152	Aquaporin PIP1-2 (*Zea mays*, PIP1-2)
GT_00029	41	0	NPR1-like protein (*Musa* ABB Group, NPR1)
GT_00030	40	0	Catalase isozyme 1 (*Gossypium hirsutum*, CATA1)
GT_00023	40	8.00E-23	DnaJ domain containing protein (*Oryza sativa* subsp. *japonica*, Q10M47_ORYSJ)

* Protein entry names and E-values were obtained from best BLASTX (nucleotide to protein) hits against the Swiss-Prot database as first choice followed by the TrEMBL database if no Swiss-Prot entry was available.

Supplemental Table S3. The most abundantly represented transcripts in specific ginger and turmeric rhizome libraries (EST number ≥ 10 per library)

Unitrans ID	EST count		Annotation	E-value	EST count of rhizome libraries*		
	From total	From rhizome			ZO_Ec	ZO_Ed	CL_Ea
Sorted by GW rhizome library (ZO_Ec)							
GT_00005	57	416	Catalase (*Musa acuminata*, Cat2)	0	34	23	0
GT_00006	46	416	S-adenosylmethionine synthetase (*Dendrobium crumenatum*, SAMS)	0	26	20	0
GT_00017	42	261	No match	24	18	0	
GT_00011	60	316	Ubiquitin (*Pisum sativum*, PUB3)	0	23	18	19
GT_00014	33	286	4-Coumarate--CoA ligase-like 1 (*Oryza sativa subsp. japonica*, 4CLL1)	1E-49	19	14	0
GT_00298	19	60	S-adenosylmethionine synthetase (*Dendrobium crumenatum*, SAMS)	0	19	0	0
GT_00101	22	110	Xyloglucan endotransglycosylase (*Musa acuminata*, XET1)	4E-143	18	4	0
GT_00205	22	75	UDP-glucose 6-dehydrogenase (*Glycine max*)	0	18	4	0
GT_00025	18	211	4-coumarate--CoA ligase-like 1 (*Oryza sativa subsp. japonica*, 4CLL1)	3E-48	18	0	0
GT_00077	18	130	Mitochondrial uncoupling protein (*Saccharum officinarum*)	1E-115	18	0	0
GT_00041	32	181	No match	16	16	0	
GT_00093	25	115	Putative MYC transcription factor (*Oryza sativa*, RAP1)	2E-29	16	9	0
GT_00088	20	120	Short-chain dehydrogenase, putative (*Ricinus communis*)	9E-106	16	4	0
GT_00249	16	65	Xyloglucan endotransglycosylase (*Musa acuminata*, XET1)	9E-138	16	0	0
Sorted by GY rhizome library (ZO_Ed)							
GT_00005	57	416	Catalase (*Musa acuminata*, Cat2)	0	34	23	0
GT_00010	31	331	Heat shock cognate 70 kDa protein (*Oryza sativa subsp. japonica*, HSP72)	0	8	23	0
GT_00055	24	155	Polyubiquitin (*Zea mays*, UBIQ)	0	3	21	0
GT_00115	24	100	Putative uncharacterized protein (*Oryza sativa subsp. japonica*)	3E-40	3	21	0
GT_00006	46	416	S-adenosylmethionine synthetase (*Dendrobium crumenatum*, SAMS)	0	26	20	0
GT_00011	60	316	Ubiquitin (*Pisum sativum*, PUB3)	0	23	18	19
GT_00017	42	261	No match		24	18	0
GT_00028	31	206	Aquaporin PIP1-2 (*Zea mays*, PIP1-2)	9E-152	13	18	0
GT_00150	29	85	Alcohol dehydrogenase, putative (*Ricinus communis*)	2E-128	11	18	0
GT_00060	24	150	Cysteine proteinase (*Sandersonia aurantiaca*, PRT22)	8E-155	6	18	0
GT_00071	18	140	Light-harvesting complex I protein (*Populus trichocarpa*, LHCA4)	6E-123	0	18	0

*Values given are normalized total EST number (TEN) ($\times 10^4$). TEN, an indicator of EST expression levels, was calculated as the sum of all ESTs that are associated with gene products carrying out a specific enzymatic activity, divided by the total EST number within a particular library.

Supplemental Table S3 (cont.). The most abundantly represented transcripts in specific ginger and turmeric rhizome libraries (EST number ≥ 10 per library)

Unitrans ID	EST count		Annotation	E-value	EST count of rhizome libraries*		
	From total	From rhizome			ZO_Ec	ZO_Ed	CL_Ea
Sorted by T3C rhizome library (CL_Ea)							
GT_00024	34	211	Polyubiquitin (*Sporobolus stapfianus*, UBIQ)	0	0	3	31
GT_00067	35	140	1-aminocyclopropane-1-carboxylate oxidase (*Musa acuminata*, MAO1B)	3E-134	0	4	31
GT_00080	31	120	Tubulin alpha-6 chain (*Zea mays*, TUBA6)	0	0	0	31
GT_00032	33	191	AMP dependent CoA ligase, putative (*Ricinus communis*)	0	0	4	29
GT_00068	28	140	S-adenosylmethionine synthetase (*Dendrobium crumenatum*, SAMS)	0	0	0	28
GT_00095	26	110	Jasmonate ZIM domain-containing protein 1 (*Arabidopsis thaliana*)	1E-27	0	0	26
GT_00160	25	85	Elongation factor 1-alpha (*Gossypium hirsutum*, EF1A5)	0	0	0	25
GT_00181	25	80	Momilactone A synthase (*Oryza sativa subsp. japonica*)	2E-68	0	0	25
GT_00094	23	110	Mitochondrial uncoupling protein (*Saccharum officinarum*)	2E-111	0	0	23
GT_00201	23	75	Farnesyl pyrophosphare synthase (*Musa acuminata*, FPPS)	4E-159	0	0	23
GT_00173	22	80	Superoxide dismutase [Cu-Zn] (*Ananas comosus*, SOD1)	2E-79	0	0	22
GT_00240	22	70	Glycosyltransferase, CAZy family GT2 (*Populus trichocarpa*)	0	0	0	22
GT_00153	20	85	Heat shock protein, putative (*Ricinus communis*)	2E-147	0	0	20
GT_00246	20	65	BURP domain-containing protein 3 (*Oryza sativa subsp. japonica*)	6E-54	0	0	20
GT_00011	60	316	Ubiquitin (*Pisum sativum*, PUB3)	0	23	18	19
GT_00075	19	130	DnaJ protein homolog 2 (*Allium porrum*, DNJH2)	0	0	0	19
GT_00113	19	100	EF-hand Ca2+binding protein CCD1 (*Zea mays*)	4E-23	0	0	19
GT_00174	19	80	Probable aquaporin PIP1-2 (*Oryza sativa subsp. japonica*, PIP1-2)	8E-149	0	0	19
GT_00040	21	181	Mitochondrial uncoupling protein (*Saccharum officinarum*)	2E-115	0	4	17
GT_00132	21	95	R2r3-myb transcription factor (*Ricinus communis*)	2E-71	0	4	17
GT_00090	19	120	Catalase (*Musa acuminata*, CAT2)	0	0	2	17
GT_00258	17	65	No match		0	0	17
GT_00364	17	55	36.4 kDa proline-rich protein (*Zea mays*)	6E-39	0	0	17
GT_00252	21	65	Shaggy-related protein kinase alpha (*Arabidopsis thaliana*)	0	5	0	16
GT_00058	20	155	AMP dependent CoA ligase, putative (*Ricinus communis*)	0	0	4	16
GT_00204	18	75	S-adenosylmethionine decarboxylase (*Malus domestica*, SAMDC)	3E-85	0	2	16
GT_00170	16	80	Mitochondrial 2-oxoglutarate/malate carrier protein (*Zea mays*)	6E-124	0	0	16
GT_00253	16	65	Probable WRKY transcription factor 40 (*Arabidopsis thaliana*)	1E-40	0	0	16

*Values given are normalized total EST number (TEN) (×10^4). TEN, an indicator of EST expression levels, was calculated as the sum of all ESTs that are associated with gene products carrying out a specific enzymatic activity, divided by the total EST number within a particular library.

Supplemental Table S4: Normalized EST expression levels for selected enzymes in ginger and turmeric metabolic pathways*

Descriptive name	EC number	Abbr.	T3C		GW			GY		
			Rh	L	Rh	L	R	Rh	L	R
**Primary/core metabolism**										
sucrose synthase	2.4.1.13	SUS	24.6	0.0	38.5	7.1	38.0	23.2	13.5	14.1
glucose-6-phosphate dehydrogenase	1.1.1.49	GPD	1.8	3.0	1.6	0.0	0.0	3.1	1.7	8.8
6-phosphogluconate dehydrogenase	1.1.1.44	6PGD	0.0	8.9	0.0	1.4	3.2	3.1	0.0	3.5
ribulose-5-phosphate isomerase	5.1.3.4	RPI	0.0	0.0	0.0	0.0	0.0	0.0	0.0	0.0
ribulose-5-phosphate epimerase	5.1.3.18	RPE	1.8	8.9	3.2	10.0	3.2	15.5	13.5	8.8
transketolase	2.2.1.1	TKT	3.5	4.4	1.6	7.1	0.0	13.9	1.7	3.5
transaldolase	2.2.1.2	TAL	3.5	0.0	6.4	2.8	3.2	7.7	6.7	5.3
pyrophosphate: fructose-6-phosphate 1-phosphotransferase	2.7.1.90	PFPP	12.3	3.0	6.4	0.0	1.6	0.0	3.4	0.0
aldolase	4.1.2.13	ADL	17.6	91.8	24.1	66.8	14.3	4.6	87.5	21.2
glyceraldehyde-3-phosphate dehydrogenase	1.2.1.12	G3P	31.7	41.5	17.7	39.8	15.8	23.2	85.8	21.2
phosphoglycerate kinase	2.7.2.3	PGK	0.0	16.3	4.8	17.1	4.8	0.0	18.5	3.5
PG mutase (phosphoglucomutase)	5.4.2.2	PGM	0.0	3.0	0.0	0.0	0.0	3.1	0.0	0.0
enolase	4.2.1.11	ENO	1.8	4.4	0.0	0.0	1.6	3.1	3.5	0.0
3-phosphoglycerate dehydrogenase	1.1.1.95	3PGD	0.0	4.4	9.6	8.5	7.9	1.5	18.5	10.6
pyruvate kinase	2.7.1.40	KPY	8.8	4.4	16.1	2.8	6.3	12.4	0.0	7.1
pyruvate dehydrogenase	1.2.1.51	PNO	1.8	3.0	6.4	5.7	6.3	3.1	10.1	3.5
acetoacetyl-CoA thiolase	2.3.1.9	AACT	54.5	22.2	22.5	46.9	14.3	40.2	16.8	17.7
**Shikimate pathway**										
DAHP synthetase	2.5.1.54	DAHPS	3.5	0.0	6.4	0.0	1.6	13.9	3.4	7.1
3-dehydroquinate synthase	4.2.3.4	DHQS	1.8	0.0	0.0	2.8	1.6	3.1	0.0	0.0
3-dehydroquinate dehydratase /shikimate dehydrogenase	1.1.1.25	DHQSD	5.3	10.4	14.4	8.5	33.3	13.9	6.7	30.1
shikimate kinase	2.7.1.71	AROK	0.0	0.0	0.0	0.0	0.0	3.1	1.7	3.5
EPSP synthase	2.5.1.19	AROA	3.5	0.0	0.0	0.0	1.6	3.1	0.0	0.0
chorismate synthase	4.2.3.5	AROC	3.5	0.0	0.0	0.0	0.0	0.0	0.0	0.0
glutamine-2-oxoglutarate aminotransferase	1.4.1.13	GOGAT	7.0	1.5	0.0	5.7	0.0	0.0	16.8	0.0
glutamine synthetase	6.3.1.2	GS	1.8	22.2	1.6	19.9	9.5	18.6	30.3	14.1
aminotransferase	2.6.1.52	AMT	3.5	0.0	0.0	0.0	0.0	9.3	0.0	0.0
**Phenylpropanoid pathway**										
phenylalanine ammonia lyase	4.3.1.5	PAL	10.6	0.0	4.8	15.6	12.7	3.1	0.0	10.6
cinnamate 4-hydroxylase	1.14.13.11	C4H	28.1	45.9	35.3	39.8	36.4	63.4	23.6	15.9
4-coumarate-CoA ligase	6.2.1.12	4CL	5.3	35.6	8.0	11.4	15.8	32.5	11.8	17.7
p-coumaroyl-CoA:shikimate *p*-coumaroyl transferase	N/A	CST	3.5	17.8	9.6	10.0	6.3	34.0	8.4	1.8
p-coumaroylshikimate-3'-hydroxylase	N/A	CS3'H	14.1	17.8	14.4	25.6	30.1	23.2	10.1	8.8
caffeoyl-CoA *O*-methyltransferase	2.1.1.104	CCOMT	14.1	0.0	14.4	7.1	4.8	1.5	3.4	7.1
**Terpenoid pathway**										
MEP pathway										
1-deoxy-D-xylulose-5-phosphate (DOXP) synthase	2.2.1.7	DXS	3.5	10.4	1.6	14.2	11.1	7.7	16.8	10.6

DOXP reductoisomerase	1.1.1.267	DXR	1.8	0.0	6.4	0.0	0.0	0.0	0.0	19.5
CDP-ME kinase	2.7.1.148	CMK	0.0	0.0	0.0	0.0	4.8	0.0	0.0	0.0
2-C-methyl-D-erythritol 2,4-cyclodiphosphate synthase	4.6.1.12	MCS	0.0	3.0	0.0	2.8	0.0	3.1	0.0	0.0
4-hydroxy-3-methylbut-2-en-1-yl diphosphate synthase	1.17.4.3	GCPE	1.8	11.9	0.0	0.0	3.2	4.6	6.7	3.5
1-hydroxy-2-methyl-butenyl 4-diphosphate reductase	1.17.1.2	HDR	0.0	0.0	3.2	2.8	0.0	1.5	3.4	0.0
4-hydroxy-3-methylbut-2-en-1-yl diphosphate reductase	1.17.1.2	IDS	0.0	3.0	3.2	7.1	0.0	1.5	10.1	0.0
MVA pathway										
HMG-CoA synthase	2.3.3.10	HMGS	0.0	0.0	0.0	0.0	6.3	0.0	0.0	0.0
HMG-CoA reductase	1.1.1.34	HMGR	0.0	0.0	0.0	0.0	0.0	0.0	0.0	3.5
MVA kinase	2.7.1.36	MVK	0.0	0.0	1.6	0.0	0.0	0.0	0.0	0.0
Common steps										
isopentenyl-diphosphate delta-isomerase	5.3.3.2	IDI	3.5	0.0	4.8	1.4	6.3	7.7	0.0	0.0
geranyl pyrophosphate synthase	2.5.1.10	FPPS	12.3	8.9	6.4	0.0	11.1	34.0	6.7	10.6
geranyl geranyl diphosphate synthase	2.5.1.29	GGPPS	6.7	0.0	3.2	1.5	7.1	4.8	3.5	8.9
terpene synthases	N/A	CS	29.9	7.4	16.1	0.0	14.3	35.6	0.0	19.5
**One carbon metabolism**										
phosphoserine phosphatase	3.1.3.3	PSP	0.0	0.0	3.2	0.0	0.0	3.1	3.4	0.0
serine hydroxymethyltransferase	2.1.2.1	GLYM	10.6	22.2	0.0	7.1	11.1	9.3	20.2	3.5
glycine dehydrogenase	1.4.4.2	GCSP	8.8	5.9	4.8	21.3	0.0	0.0	85.8	0.0
5,10-methylene-THF reductase	1.5.1.20	MTHR	3.5	0.0	3.2	4.3	0.0	0.0	0.0	3.5
5,10-methenyl-THF cyclohydrolase	3.5.4.9	FOLD	0.0	1.5	1.6	4.3	0.0	7.7	0.0	0.0
5,10-methylene-THF dehydrogenase	1.5.1.5	FOLDD	0.0	1.5	1.6	1.4	0.0	7.7	0.0	0.0
10-formyl-THF synthetase	6.3.4.3	FTHS	0.0	0.0	0.0	1.4	0.0	0.0	0.0	0.0
methionine synthase (cobalamin-independent)	2.1.1.14	METE	7.0	22.2	17.7	4.3	17.4	17.0	3.4	21.2
ATP synthase	3.6.3.14	ATPS	19.3	20.7	24.1	22.7	9.5	9.3	25.2	31.8
adenylate kinase	2.7.4.3	KAD	14.1	3.0	8.0	2.8	1.6	18.6	0.0	7.1
adenosine kinase	2.7.1.20	ADK	5.3	3.0	6.4	0.0	11.1	9.3	0.0	7.1
S-adenosylmethionine synthetase	2.5.1.6	SAM	45.7	44.4	62.6	35.5	148.9	74.2	45.4	46.0
S-adenosylhomocysteine hydrolase	3.3.1.1	SAHC	10.6	14.8	9.6	11.4	11.1	13.9	3.4	23.0

* Total normalized EST number (TEN) was determined as described for Supplemental Table S3.

Supplemental Table S5: Normalized EST expression levels for selected gene families*

Potential function (based on best hit in UniProt)	Unitrans	ESTs	T3C		GW			GY		
			Rh	L	Rh	L	R	Rh	L	R
**Polyketide synthases (PKSs)**										
type III polyketide synthase isoforms 1 and 2	20	132.7	33.7	10.0	12.8	30.9	10.6	20.6	5.3	8.9
chalcone/naregenin-chalcone synthase	4	21.6	6.8	8.5	0.0	0.0	0.0	6.3	0.0	0.0
putative PKSs	18	61.3	0.0	5.7	6.4	15.5	8.8	6.3	14.1	4.4
Total	42	206.3	33.5	32.7	19.2	39.8	14.3	32.5	18.5	15.9
**Terpene Synthases (TPSs)**										
Monoterpene										
isoprene synthase	7	22.1	3.5	0.0	0.0	0.0	0.0	17.0	0.0	1.6
limonene synthase	4	16.4	0.0	0.0	3.2	0.0	7.1	6.2	0.0	0.0
γ-terpinene synthase	3	15.3	10.5	0.0	4.8	0.0	0.0	0.0	0.0	0.0
(–)-β-pinene synthase	2	13.5	0.0	0.0	0.0	0.0	8.8	1.5	0.0	3.2
(+)-sabinene synthase	1	3.5	3.5	0.0	0.0	0.0	0.0	0.0	0.0	0.0
geraniol synthase	1	4.8	0.0	0.0	4.8	0.0	0.0	0.0	0.0	0.0
linalool synthase	1	2.9	0.0	2.9	0.0	0.0	0.0	0.0	0.0	0.0
Sesquiterpene										
germacrene d synthase	5	23.9	3.5	0.0	3.2	0.0	0.0	7.7	0.0	9.5
(+)-Δ-cadinene synthase	2	6.4	3.5	2.9	0.0	0.0	0.0	0.0	0.0	0.0
(*E*)-β-farnesene synthase	1	3.2	0.0	0.0	3.2	0.0	0.0	0.0	0.0	0.0
cascarilladiene synthase	1	1.8	0.0	0.0	0.0	0.0	1.8	0.0	0.0	0.0
patchoulol synthase	1	3.2	0.0	0.0	0.0	0.0	0.0	0.0	0.0	3.2
(*E*)-α-bergamotene synthase	1	3.5	0.0	0.0	0.0	0.0	3.5	0.0	0.0	0.0
Diterpene										
levopimaradiene synthase	2	6.6	3.5	1.5	1.6	0.0	0.0	0.0	0.0	0.0
Triterpene										
cycloartenol synthase	1	3.1	0.0	0.0	0.0	0.0	0.0	3.1	0.0	0.0
oxidosqualene cyclase	1	3.5	3.5	0.0	0.0	0.0	0.0	0.0	0.0	0.0
squalene synthase	1	1.5	0.0	0.0	0.0	0.0	0.0	1.5	0.0	0.0
Tetraterpene										
phytoene synthase	10	41.7	0.0	23.4	3.2	8.4	0.0	0.0	6.7	0.0
Total	45	177.0	31.6	30.7	24.0	8.4	21.2	37.0	6.7	17.4
**NAD(P)H-dependent Reductases/Dehydrogenases**										
10-hydroxygeraniol oxidoreductase	3	16.1	0.0	0.0	0.0	2.8	7.1	3.1	0.0	3.2
2'-hydroxyisoflavone reductase	8	27.4	1.8	0.0	0.0	2.8	8.8	6.2	0.0	7.9
3-hydroxy-3-methylglutaryl coenzyme A reductase	1	3.5	0.0	0.0	0.0	0.0	3.5	0.0	0.0	0.0
3-oxo-5-alpha-steroid 4-dehydrogenase	4	9.6	1.8	2.9	0.0	0.0	0.0	1.5	3.3	0.0
alcohol dehydrogenase	3	11.3	5.3	0.0	3.2	2.8	0.0	0.0	0.0	0.0
alcohol dehydrogenase class III	2	9.1	0.0	0.0	3.2	2.8	0.0	3.1	0.0	0.0
allyl alcohol dehydrogenase	14	78.7	21.0	5.9	14.4	11.2	10.6	13.9	1.7	0.0
anthocyanidin reductase.	2	12.3	0.0	0.0	0.0	0.0	0.0	12.3	0.0	0.0
cinnamoyl-CoA reductase	7	18.1	7.0	0.0	3.2	1.4	0.0	0.0	3.3	3.2
cinnamyl alcohol dehydrogenase	5	19.0	0.0	0.0	0.0	0.0	0.0	6.2	3.3	9.5
hydroxyphenylpyruvate reductase	1	3.3	0.0	0.0	0.0	0.0	0.0	0.0	3.3	0.0
hydroxypyruvate reductase	5	23.8	0.0	0.0	0.0	5.6	0.0	1.5	16.7	0.0
mannitol dehydrogenase	6	19.5	3.5	2.9	1.6	0.0	3.5	0.0	1.7	6.3
NAD-dependent formate dehydrogenase	2	22.8	0.0	0.0	8.0	2.8	7.1	0.0	3.3	1.6
NADPH-dependent oxidoreductase (aldo/keto)	11	39.4	0.0	8.8	3.2	12.6	7.1	6.2	0.0	1.6
phenylcoumaran benzylic ether reductase	1	2.9	0.0	2.9	0.0	0.0	0.0	0.0	0.0	0.0
progesterone 5-β-reductase	6	22.2	0.0	4.4	3.2	0.0	3.5	0.0	0.0	11.1
secoisolariciresinol	1	3.1	0.0	0.0	0.0	0.0	0.0	3.1	0.0	0.0

dehydrogenase										
short-chain alcohol dehydrogenase	36	208.5	28.1	14.6	17.6	19.6	24.7	54.0	18.3	31.6
short-chain dehydrogenase	4	13.3	8.8	0.0	0.0	1.4	0.0	0.0	0.0	3.2
sinapyl alcohol dehydrogenase	5	32.5	0.0	4.4	0.0	0.0	12.3	3.1	1.7	11.1
tropinone reductase	2	4.5	0.0	2.9	0.0	0.0	0.0	0.0	0.0	1.6
Total	129	600.9	77.2	49.8	57.7	65.8	88.2	114.1	56.6	91.6
**BAHD Acyltransferases**										
p-coumaroyl-CoA:shikimate *p*-coumaroyl transferase	28	91.4	3.5	17.8	9.6	10.0	6.3	34.0	8.4	1.8
anthocyanin 5-aromatic acyltransferase	7	27.9	0.0	11.4	0.0	0.0	3.5	9.5	3.5	0.0
3'-N-debenzoyltaxol N-benzoyltransferase	18	61.5	6.7	8.5	14.5	3.1	3.5	14.3	3.5	7.4
taxadienol acetyl transferase	8	30.6	3.4	0.0	9.6	0.0	0.0	15.8	1.8	0.0
benzoyl CoA benzyl alcohol benzoyl transferase	4	14.5	0	0	0	4.6	0	6.3	0	0
anthranilate N-benzoyltransferase protein	1	3.4	3.4	0.0	0.0	0.0	0.0	0.0	0.0	0.0
EIG-I24 protein	1	3.5	0.0	0.0	0.0	0.0	0.0	0.0	3.5	0.0
N-hydroxycinnamoyl/benzoyltransferase	9	32.2	3.4	5.7	6.4	7.7	0.0	1.6	0.0	7.4
putative alcohol acetyltransferase	2	8.6	0.0	0.0	0.0	1.5	0.0	0.0	7.0	0.0
acetylglucosamine acyltransferase	6	21.5	1.7	0.0	3.2	0.0	7.1	9.5	0.0	0.0
lecithine cholesterol acyltransferase	2	8.6	0.0	0.0	0.0	1.5	0.0	0.0	7.0	0.0
glyoxysomal beta-ketoacyl-thiolase	8	35.3	3.4	0	0.0	13.9	3.5	1.6	7.0	5.9
similarity to lysophosphatidic acid acyltransferase	3	6.3	0.0	0.0	0.0	3.1	0.0	3.2	0.0	0.0
putative mono-or diacylglycerol acyltransferase	2	8.8	0.0	5.7	0.0	3.1	0.0	0.0	0.0	0.0
glycerol-3-phosphate acyltransferase	7	20.4	1.7	2.8	3.2	6.2	1.8	4.8	0.0	0.0
diacylglycerol acyltransferase	1	3.2	0.0	0.0	0.0	0.0	0.0	3.2	0.0	0.0
glucose acyltransferase	14	38.6	5.0	5.7	8.0	3.1	5.3	3.2	5.3	3.0
10-deacetylbaccatin III-10-O-acetyl transferase	4	19.2	0.0	0.0	8.0	3.1	3.5	1.6	0.0	3.0
fatty acid elongase	2	6.5	0.0	0.0	0.0	0.0	3.5	0.0	0.0	3.0
taxadien-5-alpha-ol O-acetyltransferase	1	3.5	0.0	0.0	0.0	0.0	3.5	0.0	0.0	0.0
Total	128	445.5	32.2	57.6	62.5	60.9	41.5	108.6	46.6	31.5
**2-Oxoglutarate-Dependent Dioxygenases**										
4,5-DOPA dioxygenase extradiol	7	41.5	3.4	2.8	9.6	3.1	10.6	1.6	0.0	10.4
lipoxygenase	30	115.5	11.8	5.7	14.4	26.3	12.4	9.5	8.8	26.7
acireductone dioxygenase	8	36.6	5.0	1.4	8.0	18.6	0.0	0.0	3.5	0.0
putative lipoxygenase	9	22.2	1.7	5.7	4.8	1.5	1.8	3.2	3.5	0.0
9-cis-epoxy-carotenoid dioxygenase	4	18.7	0.0	4.3	1.6	0.0	0.0	11.1	1.8	0.0
4-hydroxyphenylpyruvate dioxygenas (4HPPD)	2	4.4	0.0	2.8	0.0	0.0	0.0	1.6	0.0	0.0
β-carotene dioxygenase	4	13.5	6.7	1.4	0.0	0.0	5.3	0.0	0.0	0.0
leucoanthocyanidin dioxygenase	6	28.0	0.0	5.7	0.0	3.1	0.0	14.3	3.5	1.5
ethylene-forming-enzyme-like dioxygenase	5	17.6	0.0	8.5	0.0	0.0	0.0	3.2	0.0	5.9
1-aminocyclopropane-1-carboxylate oxidase	21	135.6	6.7	21.3	6.4	1.5	10.6	76.0	7.0	5.9
2-oxoglutarate-dependent dioxygenase (2ODD)	3	9.8	3.4	0.0	6.4	0.0	0.0	0.0	0.0	0.0

putative dioxygenase	20	71.2	5.0	5.7	8.0	23.2	5.3	9.5	7.0	7.4
Total	119	514.6	43.7	65.3	59.2	77.3	46	130	35.1	57.8
**SABATH Carboxylmethyltransferases**										
salicylic acid carboxylmethyltransferase	4	17.1	6.7	2.8	0.0	0.0	0.0	1.6	0.0	5.9
carboxyl methyltransferase	2	6.2	0.0	0.0	0.0	6.2	0.0	0.0	0.0	0.0
Total	6	23.3	6.7	2.8	0	6.2	0	1.6	0	5.9
**Small Molecule *O*-methyltransferases SMOMTs**										
orcinol *O*-methyltransferase	8	31.1	5.0	4.3	8.0	0.0	0.0	6.3	0.0	7.4
caffeic acid *O*-methyltransferase	6	20.5	3.4	0.0	8.0	3.1	0.0	1.6	0.0	4.4
flavonoid *O*-methyltransferase	2	3.3	1.7	0.0	0.0	0.0	0.0	1.6	0.0	0.0
caffeoyl-CoA *O*-methyltransferase	6	52.4	14.1	0.0	14.4	7.1	4.8	1.5	3.4	7.1
Total	22	107.3	24.2	4.3	30.4	10.2	4.8	11.0	3.4	18.9

* Total normalized EST number (TEN) was determined as described for Supplemental Table S3.

Supplemental Table S6: Normalized EST expression levels for cytochrome P450 monooxygenases*

Potential function (based on best hit in UniProt)	From Tree	Unitrans	EST	T3C		GW			GY		
				Rh	L	Rh	L	R	Rh	L	R
allene oxide synthase	CYP74A group1	10	41	0	2.9	3.2	9.8	0	20	5	0
	CYP74A group2	5	17	0	0	1.6	1.4	0	12	1.7	0
fatty acid hydroperoxide lyase	CYP74F	1	3.1	0	0	0	0	0	3.1	0	0
ω-hydroxylase for fatty acids[#]	CYP86A	1	3.5	0	0	0	0	3.5	0	0	0
obtusifoliol-14-demethylase	CYP51	1	1.6	0	0	0	0	0	0	0	1.6
	CYP51G	7	24.2	1.8	0	4.8	0	0	3.1	6.7	7.9
berbamunine synthase	CYP80	1	1.4	0	0	0	1.4	0	0	0	0
carotenoid e-ring hydroxlation	CYP97A	1	1.6	0	0	0	0	0	0	0	1.6
	CYP97B	1	1.5	0	1.5	0	0	0	0	0	0
N-demethylase or ring-methyl hydroxylase	CYP71A group1	2	4.5	0	2.9	1.6	0	0	0	0	0
flavonoid 3'-hydroxylase	CYP75B	3	9.4	0	1.5	6.4	0	0	1.5	0	0
flavonoid 3' 5'-hydroxylase	CYP706C	1	2.8	0	0	0	2.8	0	0	0	0
	CYP75A	2	6.7	3.5	0	3.2	0	0	0	0	0
cinnamate-4-hydroxylase	CYP73A	5	29.2	0	2.9	0	5.6	3.5	9.3	0	7.9
p-coumaroyl shikimate 3'-hydroxylase	CYP98A	6	28.6	0	1.5	0	5.6	7.1	1.5	6.7	6.3
hydroxylation of indole to benzoxazinones (Bxs)	CYP71C	8	28.9	0	7.3	3.2	8.4	3.5	0	3.3	3.2
8'-hydroxylase for ABA	CYP707A	3	9.4	0	5.9	0	0	3.5	0	0	0
limonene hydroxylase	CYP71D	13	80.7	7	10	6.4	2.8	8.8	28	5	13
ent-kaurene oxidase	CYP701A	2	5	1.8	0	3.2	0	0	0	0	0
ent-kaurenoic acid oxidase	CYP88A	1	2.8	0	0	0	2.8	0	0	0	0
unkown	CYP703A	5	22.9	11	2.9	6.4	0	0	3.1	0	0
	CYP704A	4	9.9	3.5	0	4.8	0	0	0	0	1.6
	CYP71 group1[†]	7	25.9	7	0	4.8	0	0	7.7	0	6.3
	CYP71 group2	1	3.5	0	0	0	0	3.5	0	0	0
	CYP714B	1	3.2	0	0	3.2	0	0	0	0	0
	CYP714C	1	3.1	0	0	0	0	0	3.1	0	0
	CYP71A group2	1	3.2	0	0	3.2	0	0	0	0	0
	CYP71G	3	6.3	0	1.5	0	0	0	1.5	3.3	0
	CYP71J	3	9.2	0	0	0	1.4	0	6.2	1.7	0
	CYP71P	2	3.2	1.8	0	0	1.4	0	0	0	0
	CYP71R	3	4.5	0	1.5	0	1.4	0	0	1.7	0
	CYP71T	4	8	0	2.9	0	0	3.5	1.5	0	0
	CYP722A	1	1.8	1.8	0	0	0	0	0	0	0
	CYP72A	32	119	8.8	13	1.6	32	8.8	6.2	20	28
	CYP734A	1	1.6	0	0	0	0	0	0	0	1.6
	CYP77A	1	2.8	0	0	0	2.8	0	0	0	0
	CYP78A	1	1.6	0	0	1.6	0	0	0	0	0
	CYP89B group1	3	7.9	0	1.5	0	0	0	3.1	3.3	0
	CYP89B group2	4	12.2	0	2.9	0	2.8	0	0	3.3	3.2
	CYP92A	7	23.7	0	0	0	13	0	0	0	11
	CYP94B	3	11.7	0	0	1.6	0	7.1	3.1	0	0
	CYP94C	2	9.3	0	0	0	0	0	9.3	0	0
	CYP94D	2	5.7	0	2.9	0	2.8	0	0	0	0
	CYP96B group1	4	15	8.8	2.9	0	0	0	0	3.3	0
Total		170	618	56	69	61	98	53	123	65	93

* Total EST number (TEN) was determined as described for Supplemental Table S3.

[#] ω-hydroxylase for fatty acids: ω-hydroxylase for saturated and unsaturated C12 to C18 fatty acids.

[†] CYP71 group1 clade is adjacent to the CYP99A clade, while CYP71 group2 clade is adjacent to CYP71K, X ,Y clades as illustrated in **Supplemental Figure S4**).

**Supplemental Table S7:** Normalized percentage of ArREST ESTs with GO associations				
		Normalized EST abundance (%)		
GO	description	ArREST ESTs	Rhizome ESTs	Rhizome enriched
0006464	protein modification	10.42	11.97	24.72
0006350	transcription	8.51	9.16	12.19
0006412	protein biosynthesis	6.72	7.06	9.34
0006810	transport	9.15	9.10	5.83
0008152	metabolism	9.02	8.29	7.40
0009987	cellular process	7.96	8.41	7.08
0006118	electron transport	5.64	5.47	6.95
0000004	biological process unknown	5.31	5.40	1.51
0009058	biosynthesis	4.16	4.32	4.13
0019538	protein metabolism	3.93	4.29	3.71
0005975	carbohydrate metabolism	3.55	3.66	2.57
0015979	photosynthesis	3.46	0.71	0.18
0016043	cell organization and biogenesis	2.91	3.32	2.90
0006139	nucleotide and nucleic acid metabolism	2.50	2.77	1.08
0006091	generation of precursor metabolites and energy	2.72	1.27	0.48
0006519	amino acid and derivative metabolism	2.09	1.98	0.76
0007165	signal transduction	1.60	2.09	1.74
0006950	response to stress	1.86	1.93	1.15
0006629	lipid metabolism	1.43	1.57	1.61
0009056	catabolism	1.54	1.49	0.80
0009628	response to abiotic stimulus	1.45	1.44	1.21
0006259	DNA metabolism	0.99	1.05	0.80
0007582	physiological process	0.55	0.51	0.17
0007049	cell cycle	0.32	0.44	0.05
0019748	secondary metabolism	0.28	0.26	0.42
0009607	response to biotic stimulus	0.36	0.39	0.28
0008219	cell death	0.24	0.32	0.15
0009719	response to endogenous stimulus	0.26	0.31	0.12
0008150	biological_process	0.22	0.25	0.30
0007275	development	0.25	0.24	0.00
0019725	cell homeostasis	0.23	0.22	0.06
0030154	cell differentiation	0.05	0.05	0.18
0009605	response to external stimulus	0.09	0.08	0.00
0000003	reproduction	0.04	0.04	0.04
0009790	embryonic development	0.03	0.04	0.04
0009835	ripening	0.04	0.02	0.04
0009653	morphogenesis	0.02	0.01	0.00
0009991	response to extracellular stimulus	0.01	0.02	0.00
0009606	tropism	0.02	0.01	0.00
0009791	post-embryonic development	0.01	0.02	0.00
0040029	regulation of gene expression, epigenetic	0.01	0.01	0.00
0009908	flower development	0.01	0.01	0.00
0016049	cell growth	0.01	0.01	0.00
0009856	pollination	0.00	0.01	0.00
0007267	cell-cell signaling	0.00	0.00	0.00

Normalized EST abundance = (Σ GO contribution)/(total # GO classified ESTs in dataset). GO contribution = (# ESTs comprising a particular unitrans)/(# GO identifiers for a particular unitrans).

Supplemental Table S8: Probable transcriptional regulator classes within ArREST associated with GO:0003677

TYPE	ArRESTs		ZO_CL_Rh		CL_Eb		ZO_Ec_Ed		ZO_Ec		ZO_Ed	
	Unitrans	ESTs	Unitrans	ESTs	Unitrans	ESTs	Unitrans	ESTs	Unitrans	ESTs	Unitrans	ESTs
ARF/Aux/IAA	21	42	8	15	5	10	3	5	2	3	1	2
B3	13	23	6	9	4	5	2	4	0	0	2	4
bHLH	12	33	8	17	3	9	5	8	4	6	1	2
bZIP	84	150	41	58	12	18	29	40	19	25	10	15
CONSTANS	46	128	19	32	1	2	18	30	7	10	11	20
ERF	21	61	14	24	3	6	11	18	7	10	4	8
HD_ZIP	40	95	20	37	5	12	15	25	5	8	10	17
GRAS	2	3	0	0	0	0	0	0	0	0	0	0
HOMEOBOX	74	117	31	39	7	9	24	30	12	13	12	17
HSF	24	41	11	15	4	5	7	10	4	5	3	5
MADS	8	16	6	10	1	2	5	8	3	5	2	3
MYB	151	346	80	139	33	61	47	78	23	36	24	42
NAC	86	262	45	78	9	21	36	57	14	20	22	37
SAND	1	2	1	2	1	2	0	0	0	0	0	0
WD40	3	7	1	1	0	0	1	1	1	1	0	0
WRKY	96	241	55	110	23	52	32	58	16	29	16	29
Znf_AN1	29	116	22	50	6	13	16	37	14	31	2	6
Znf_BED	2	11	1	2	1	2	0	0	0	0	0	0
Znf_C2H2	8	23	4	9	0	0	4	9	3	7	1	2
Znf_C5HC2	3	3	1	1	1	1	0	0	0	0	0	0
Znf_CCCH	18	30	10	14	4	7	6	7	5	6	1	1
Znf_CW	3	6	1	2	0	0	1	2	1	2	0	0
Znf_DOF	12	27	7	11	3	5	4	6	4	6	0	0
Znf_GATA	34	74	20	39	4	10	16	29	8	16	8	13
Znf_PHD	7	14	4	5	0	0	4	5	2	2	2	3
Znf_RING	2	2	0	0	0	0	0	0	0	0	0	0
Znf_SBP	19	36	10	13	1	1	9	12	6	9	3	3
Total	819	1909	426	732	131	253	295	479	160	250	135	229
