# Supplementary material for: Ginger and turmeric expressed sequence tags identify signature genes for rhizome identity and development and the biosynthesis of curcuminoids, gingerols and terpenoids
Source: BMC Plant Biol. 2013 Feb 15;13:27. doi: 10.1186/1471-2229-13-27 (PMC3608961; doi:10.1186/1471-2229-13-27)
Supplement: Additional file 2: Figure S1 — Distribution of EST sequence length. The average EST sequence length was 817 bp, with unitrans (unique transcripts, a.k.a. contigs) lengths ranging from 151 to 4021 bp, with the greatest number of unitrans having between 701 and 800 bp, and with 95% exceeding 300 bp. Figure S2. Gene Ontology (GO) annotations of ArREST unitrans. 87.6% of the ArREST unitrans could be annotated by GO classification. Eight GO categories had EST abundances greater than 5%, including protein modification (10.5%), transport (9.2%), metabolism (9.0%), transcription (8.6%), cellular process (8.0%), protein biosynthesis (6.9%), electron transport (5.6%), and biological process unknown (5.3%). In contrast, the GO category secondary metabolism contained relatively few ESTs (0.3%). Figure S3. Proposed metabolic map showing how the curcuminoids, gingerols and terpenoids are produced from sucrose in a large interconnected network. Names of enzymes identified in the ArREST database are colored blue; % values indicate fraction of unitrans in the database that are represented by genes encoding each protein. Figure S4. Phylogenetic tree of P450 monooxygenases. A neighbor joining tree was generated with 1136 P450s including 170 ginger and turmeric P450s from the ArREST database (indicated by black diamonds), 247 Arabidopsis P450s, 350 rice P450s and 369 P450s from other plants. P450 subfamily classifications are indicated. Additional file1: Table S6 contains a summary of these data for ginger and turmeric. As the tree is so large, it was impossible to display it at readable scale on a page size that is typical. Thus, details of tree can been seen by zooming in on this page of the PDF file. Figure S5. Overall gene expression in rhizomes is similar to but distinct from that observed for other plant tissues. Total ArREST ESTs, ESTs with shared expression in ginger or turmeric rhizomes and at least one other ginger or turmeric tissue, and ESTs found exclusively in ginger or turmeric rhizomes are represente [file 1471-2229-13-27-S2.pdf]

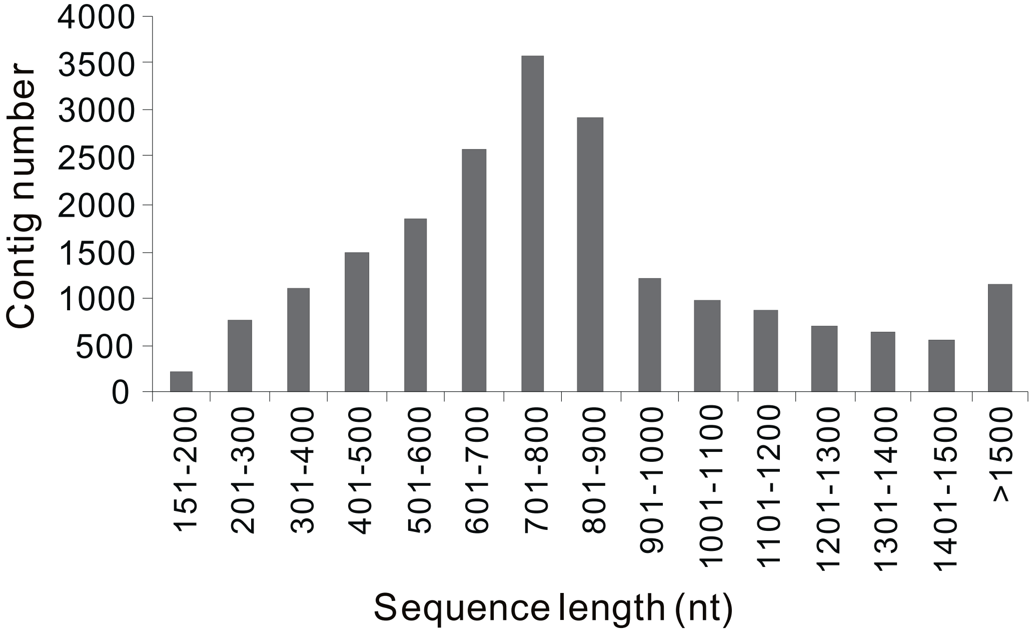

Supplemental Figure S1 Distribution of EST sequence length. The average EST sequence length was 817 bp, with unitrans (unique transcripts, a.k.a. contigs) lengths ranging from 151 to 4021 bp, with the greatest number of unitrans having between 701 and 800 bp, and with 95% exceeding 300 bp

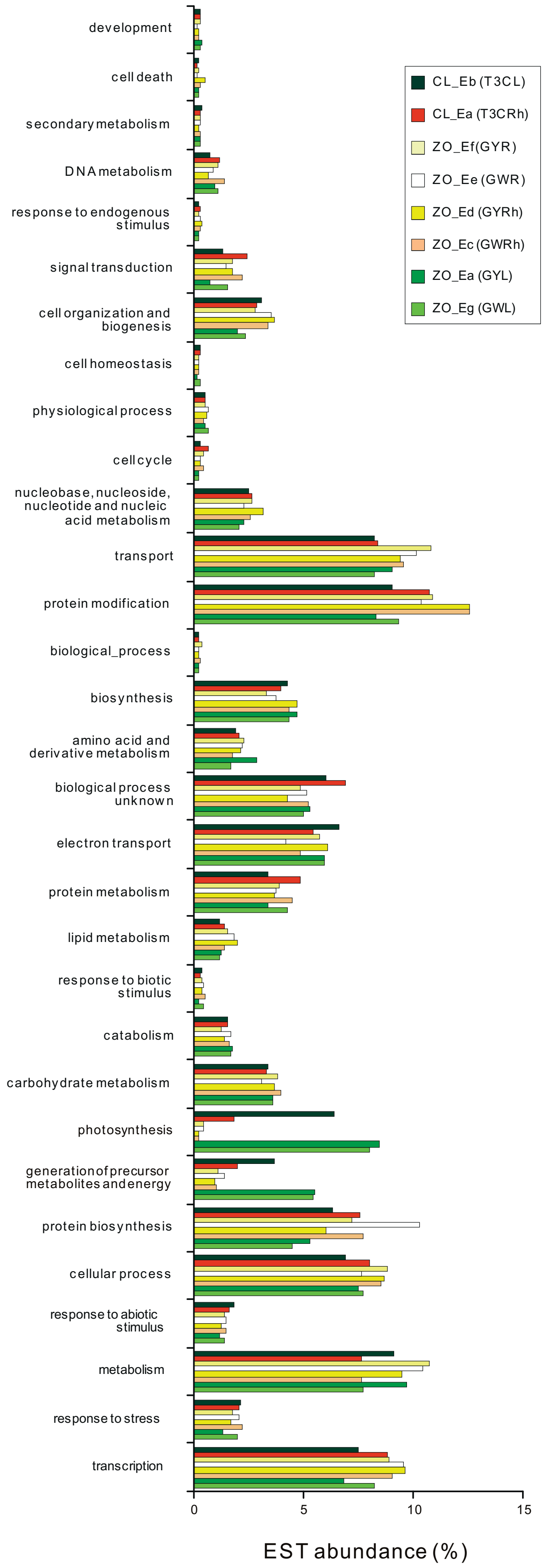

Supplemental Figure S2
Gene Ontology (GO) annotations of ArREST unitrans. 87.6 % of the ArREST unitrans could be annotated by GO classification. Eight GO categories had EST abundances greater than 5%, including protein modification (10.5%), transport (9.2%), metabolism (9.0%), transcription (8.6%), cellular process (8.0%), protein biosynthesis (6.9%), electron transport (5.6%), and biological process unknown (5.3%). In contrast, the GO category secondary metabolism contained relatively few ESTs (0.3 %).

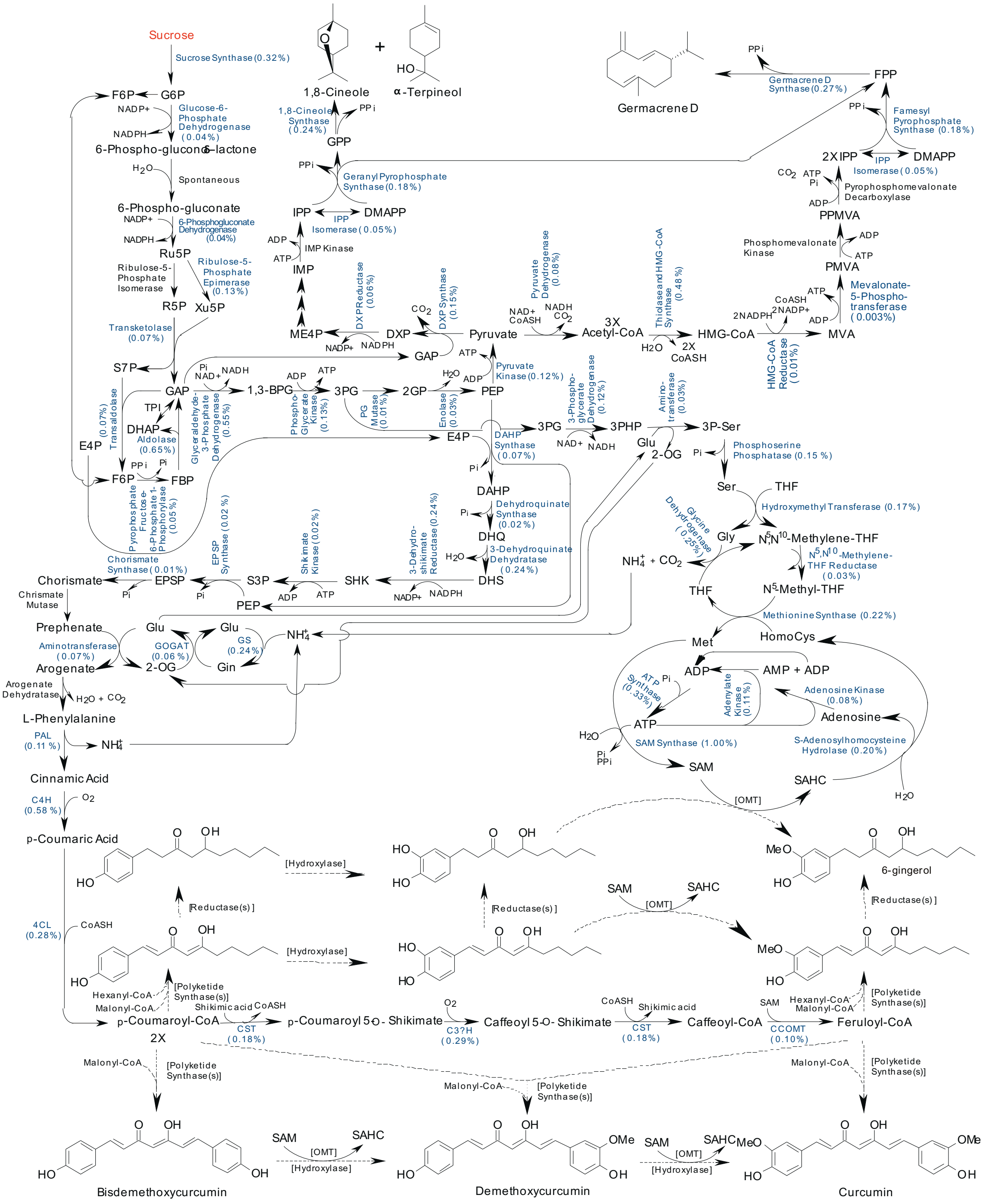

Supplemental Figure S3 Proposed metabolic map showing how the curcuminoids, gingerols and terpenoids are produced from sucrose in a large interconnected network. Names of enzymes identified in the ArREST database are colored blue; % values indicate fraction of unitrans in the database that are represented by genes encoding each protein.

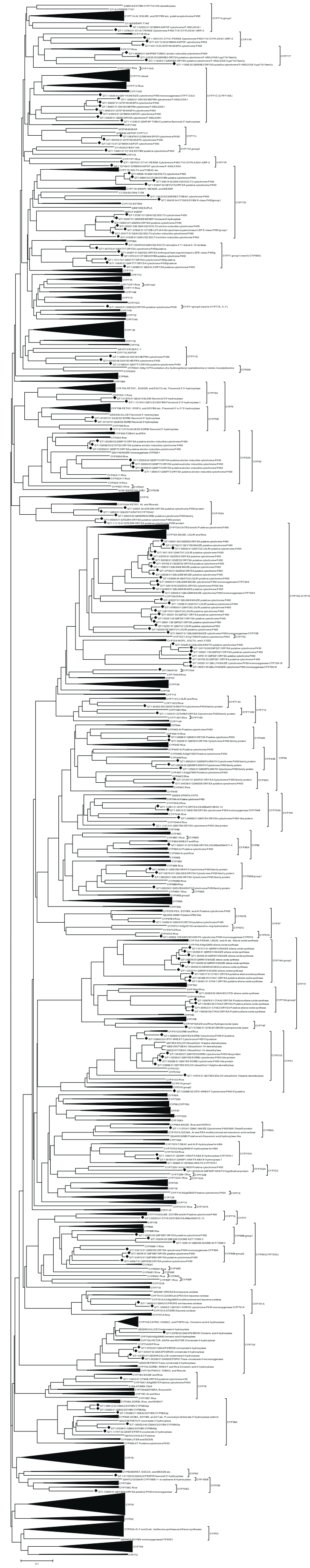

Supplemental Figure S4 Phylogenetic tree of P450 monooxygenases. A neighbor joining tree was generated with 1136 P450s including 170 ginger and turmeric P450s from the ArREST database (indicated by black diamonds), 247 Arabidopsis P450s, 350 rice P450s and 369 P450s from other plants. P450 subfamily classifications are indicated.
* Additional file 1: Supplemental Table S6 contains a summary of these data for ginger and turmeric.
* As the tree is so large, it was impossible to display it at readable scale on a page size that is typical. Thus, details of tree can been seen by zooming in on this page of the PDF file.

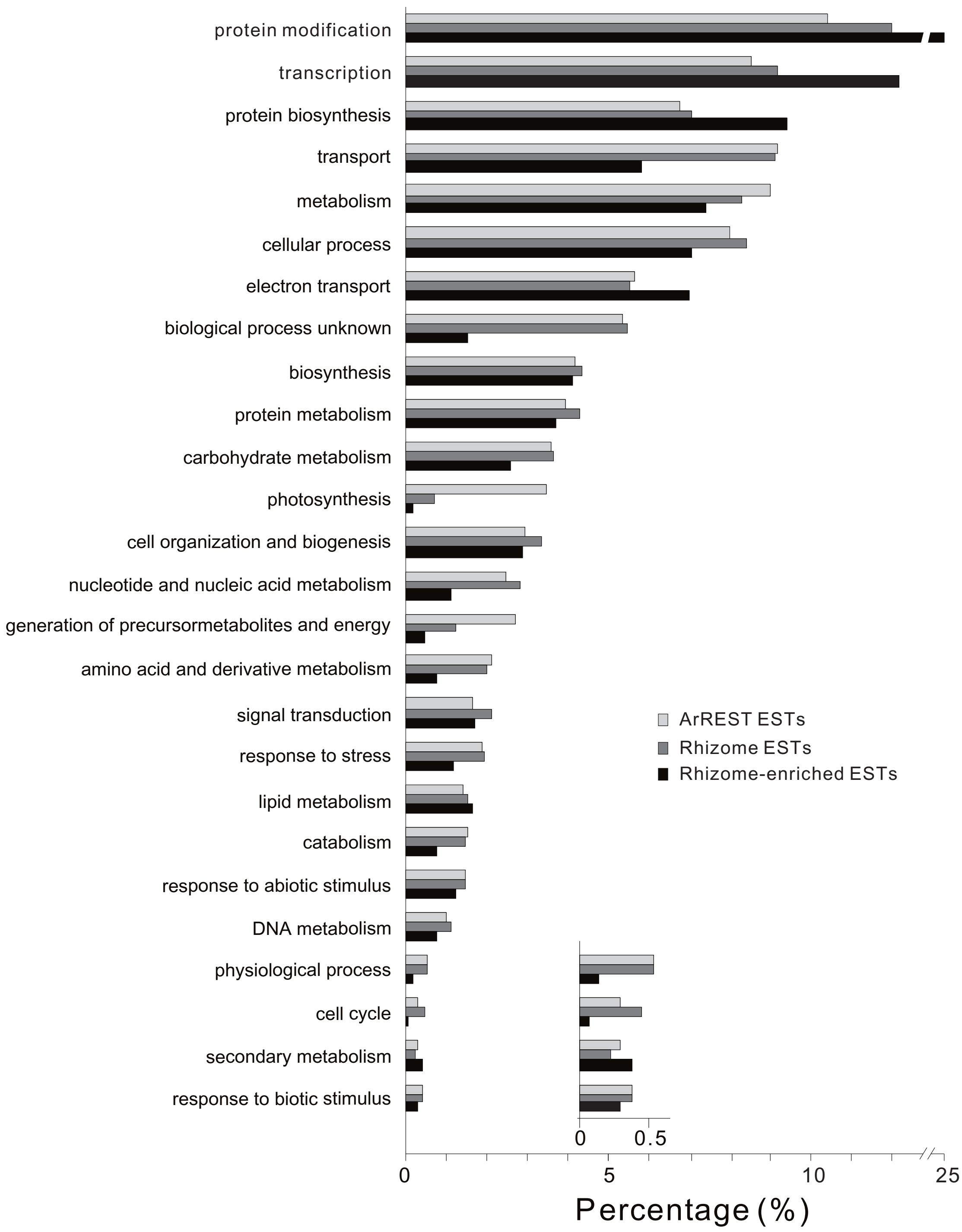

Supplemental Figure S5 Overall gene expression in rhizomes is similar to but distinct from that observed for other plant tissues. Total ArREST ESTs, ESTs with shared expression in ginger or turmeric rhizomes and at least one other ginger or turmeric tissue, and ESTs found exclusively in ginger or turmeric rhizomes are represented as light grey, dark grey and black bars, respectively. Values used to generate this graph are presented in Additional file 1: Supplemental Table S7.
