# Supplementary material for: Ginger and turmeric expressed sequence tags identify signature genes for rhizome identity and development and the biosynthesis of curcuminoids, gingerols and terpenoids
Source: BMC Plant Biol. 2013 Feb 15;13:27. doi: 10.1186/1471-2229-13-27 (PMC3608961; doi:10.1186/1471-2229-13-27)
Supplement: Additional file 3 — GenBank Accessions for Rhizome-specific Transcripts from Sorghum. [file 1471-2229-13-27-S3.pdf]

GenBank Accessions for Rhizome-specific Transcripts from Sorghum

GI61115604 - GI61115077,

GI61099298 - GI61099027,

GI56932432GI54413984, GI54413982, GI54413980, GI54413965, GI54413963, GI54413961, GI54413959,GI54413957, GI54413955, GI54413953, GI54413951, GI54413949, GI54413946, GI54413944,GI54413942, GI54413940, GI54413938, GI54413936, GI54413934, GI54413932, GI54413930,GI54413928, GI54413926, GI54413924, GI54413922, GI54413920, GI54413918, GI54413916,GI54413914, GI54413912, GI54413910, GI54413908, GI54413906, GI54413904, GI54413902,GI54413900, GI54413898, GI54413896, GI54413894, GI54413892, GI54413890, GI54413888,GI54413886, GI54413884, GI54413882, GI54413880, GI54413878, GI54413876, GI54413874,GI54413872, GI54413870, GI54413868, GI54413865, GI54413863, GI54413861, GI54413859,GI54413857, GI54413855, GI54413853, GI54413851, GI54413849, GI54413847, GI54413845,GI54413844, GI54413842, GI54413840, GI54413839, GI54413837, GI54413835, GI54413833,GI54413831, GI54413829, GI54413827, GI54413825, GI54413823, GI54413821, GI54413818,GI54413815, GI54413813, GI54413811, GI54413809, GI54413807, GI54413805, GI54413803,GI54413801, GI54413795,

GI31072290 – GI31072275,

GI21998059 – GI21788249.
