# Supplementary material for: Ginger and turmeric expressed sequence tags identify signature genes for rhizome identity and development and the biosynthesis of curcuminoids, gingerols and terpenoids
Source: BMC Plant Biol. 2013 Feb 15;13:27. doi: 10.1186/1471-2229-13-27 (PMC3608961; doi:10.1186/1471-2229-13-27)
Supplement: Additional file 4 — Microarray experimental methods, oligonucleotide probe design, microarray design (interwoven design). [file 1471-2229-13-27-S4.pdf]

Oligonucleotide probe design (summary)

From 20599 unitrans (contigs) from ginger and turmeric libraries (using the GT1 database, which is an earlier version of the database compared to the GT database now online), we designed 45 - 60-mer oligonucleotide probes in Tm range of 73.5 – 83.5 ℃ using Agilent eArray (https://earray.chem.agilent.com/earray/). For the unitrans that have homology with other genes and have orientation information, 17473 probes were prepared. For the unitrans without orientation information, we designed probes in both directions, which included 2935 and 2928 probes.

Oligonucleotide probe design (detailed)

All of the GT1 unitrans were blasted against the UniProt/TrEMBL database by Blastx with an E-value cut off of e-5. From 20599 unitrans blasted, 2935 unitrans had no hits whereas 17663 unitrans had blast hits, among which 10395 unitrans were in the correct orientation and 7268 were in the reverse orientation. The reverse complementary sequences of these 7268 unitrans were used to designed microarray oligos. The GT1 unitrans were from a previous version of the ginger/turmeric EST database. The newer version of the database, called now simply GT, is almost identical to the GT1 database, but because it was processed using slightly more stringent parameters and was annotated more recently, the GT database was made available to the public at our website.

Processing of 17633 unitrans with blast hits

We used Agilent eArray (https://earray.chem.agilent.com/earray/) to design microarray oligo sequences without the 3´ Bias option with oligo lengths of ~ 60 bases. For the 17663 unitrans that we know the orientation, we designed microarray oligos in the sense orientation of each gene sequence. After eArray processing, oligos were designed for 17650 unitrans, of which 17068 were in the Tm range of 73.5 – 83.5 ℃, and 582 unitrans were not. The eArray results indicate whether an oligo from one unitrans can be hybridized with other unitrans or not, which is referred to as x-hybridization. From 17068 oligos in the desirable Tm range, 13115 oligos had no x-hybridization with other unitrans, while 3953 oligos could be x-hybridized with other unitrans. The 13115 oligos without x-hybridization could be used as microarray oligos directly without further processing.

Of the 582 unitrans that could not produce oligos of the desirable Tm range, 573

were able to produce oligos in the Tm range of 73.5-83.5 by application of several different options: Tm range from 73.5 – 83.5 °C with trim (trim option makes the oligo length variable from 45 to 60 bases), Tm set at 78.5 °C without trim with fixed oligo length of 45 bases, Tm set at 78.5 °C without trim with fixed oligo length of 50 bases, Tm set at 78.5 °C without trim with fixed oligo length of 55 bases, and Tm set at 78.5 °C without trim with fixed oligo length of 60 bases. All the resulting oligo sequences were sorted first by no x-hybridization and then by higher oligo length because we were more concerned with having less x-hybridization than longer length oligos. Thus, 573 oligos were found with Tm range 73.5 – 83.5 °C and an average oligo length of 57.5 bases.

For the 3953 oligos that had potential hybridization problems with other unitrans, we blasted them against the 17663 GT1 unitrans in the correct orientation using Blastn with an E-value cut off of e-5. We also blasted the 573 oligos that were then in the desirable Tm range against the 17663 GT1 unitrans by Blastn as above. This was to determine which oligos were specific to one unitrans, or could also potentially cross hybridize. Because of the nature of the original EST database, it was possible that the identical sequence could exist in two separate unitrans because these failed to assemble.

We merged these blast data and sorted the oligos in the order of higher x-hybridization number to other unitrans. In this format, for example, the oligo listed in the first line can be used for 32 unitrans including its own unitrans and the second line oligo is for 13 unitrans and the last line oligo is for only its own unitrans. We removed oligos from further consideration that were already x-hybridized with oligos listed above and that had also exactly the same oligo sequence with them. After this processing, 150 oligos were removed from the list and 4358 oligos remained.

Finally 4358 oligos processed for optimal Tm and removal of duplicated oligos and 13115 oligos gathered in the initial step combined to make 17473 microarray oligos.

Process of 2935 unitrans without blast hit

For 2935 unitrans without orientation information, we processed them in the same way that we processed the unitrans with orientation information. However these unitrans were processed in both sense and antisense orientation separately because we did not know their orientation. 2928 oligos were identified when they were considered in the sense orientation and 2929 oligos in the antisense orientation.

Microarray design: Interwoven design

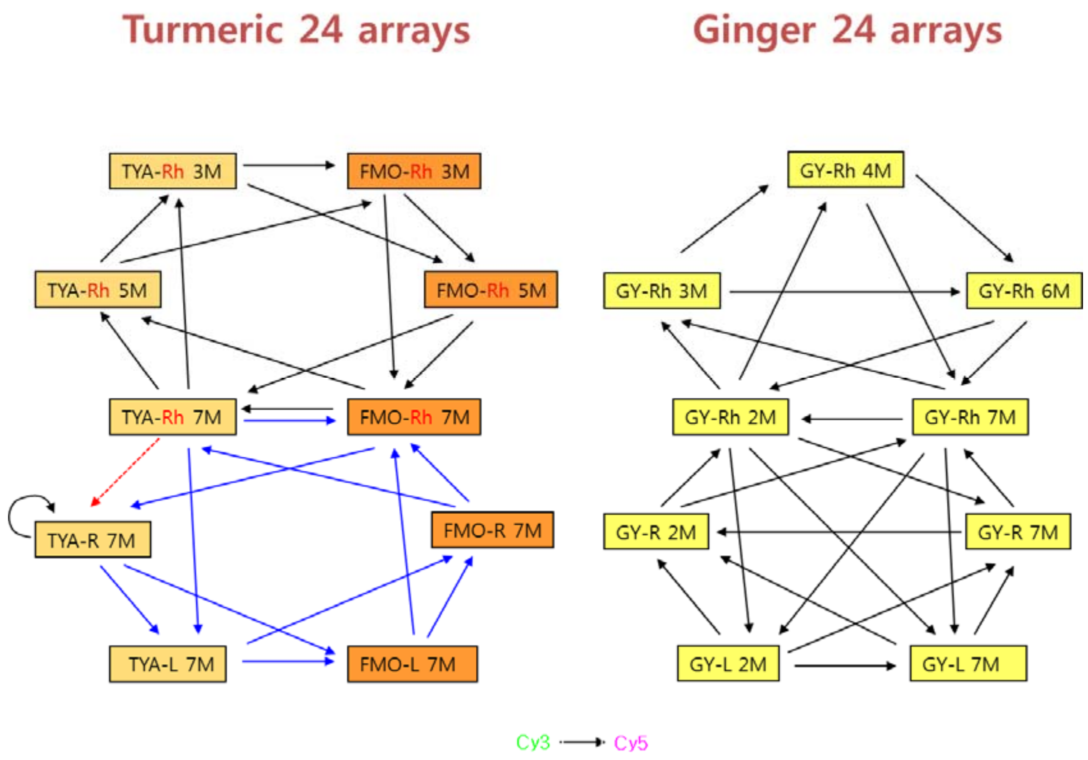

Red dotted arrow (TYA-Rh 7M to TYA-R 7M): original design

Black rounded arrow (TYA-R 7M): actual experiment

Microarray experiments

Microarray printing

For microarray printing of Agilent 4x44K format slides, 17,473 probes with known orientation information were printed twice and 3000 probes with higher x-hybridization numbers from 17,473 probes were printed once. Probes from unitrans without orientation information, 2928 Probes in sense orientation and 2929 oligos in antisense orientation were printed once. In total, 43,803 spots were printed in each array.

After we performed initial 4x44K format microarray experiments with ginger and turmeric samples, we reduced probe numbers to 15208, based on consideration of spot intensity and reproducibility. Probes with very low intensity across all ginger and turmeric tissue samples were excluded. Also probes with very high standard deviations in replicate hybridizations were excluded.

This allowed us to use the Agilent 8x15K format instead, with 15208 probes used.

Target preparation

Plant tissues were harvested each month and stored at -80 ℃ until RNA extraction. Total RNA was extracted using the RNeasy Plant Mini Kit (Qiagen) according to the manufacturer's protocol. From 1 ug of total RNA, aRNA was prepared using the MessageAmp II aRNA Amplification Kit (Ambion) according to the manufacturer's protocol. 4 ug of aRNA was dried using a Speedvac centrifuge at room temperature and labeled with Cy3 or Cy5 (GE Healthcare) according to the microarray target preparation and hybridization method (http://cals.arizona.edu/microarray/methods.html). Hybridization samples were prepared according to the protocol provided by Agilent (http://www.chem.agilent.com/Library/usermanuals/Public/G4140-90050_Two-Color_GE_5.7.pdf) using Cy3 or Cy5 labeled aRNA, 10X Blocking Agent (Agilent), 25X Fragmentation Buffer (Agilent) and 2x GEx Hybridization Buffer HI-RPM (Agilent).

Hybridization

We used corresponding gaskets for either 4x44K or 8k15K slides and Agilent Microarray Hybridization Chamber Kit to seal the hybridization buffer inside the gasket. Assembled slide chambers in rotisserie in a 65 ℃ hybridization oven were rotated at 10 rpm for 17 hours. We washed the slides using GE Wash Buffer 1 (Agilent), GE Wash Buffer 2 (Agilent), Acetonitrile (Burdick & Jackson) and Stabilization and Drying Solution (Agilent) according to the protocol provided by Agilent (http://www.chem.agilent.com/Library/usermanuals/Public/G4140-90050_Two-Color_GE_5.7.pdf).

Data acquisition and analysis

The microarray slides were scanned with a Gene Pix 4000B scanner (Axon/Molecular Devices) at a resolution of 5 µm per pixel with laser illumination (100% power) at 532 and 635 nm with PMT automatically adjusted at 0.01% saturation. From TIFF file, spot findings and data extraction was done using Agilent Feature Extraction Software (Agilent)
